# Simulating CXCR5 Dynamics in Complex Tissue Microenvironments

**DOI:** 10.3389/fimmu.2021.703088

**Published:** 2021-09-07

**Authors:** Jason Cosgrove, Kieran Alden, Jens V. Stein, Mark C. Coles, Jon Timmis

**Affiliations:** ^1^Department of Electronic Engineering, University of York, York, United Kingdom; ^2^Institut Curie, Université PSL, Sorbonne Université, CNRS UMR168, Laboratoire Physico Chimie Curie, Paris, France; ^3^Department of Oncology, Microbiology and Immunology, University of Fribourg, Fribourg, Switzerland; ^4^Kennedy Institute of Rheumatology at the University of Oxford, Oxford, United Kingdom; ^5^School of Computer Science, University of Sunderland, Sunderland, United Kingdom

**Keywords:** B cells, chemokines, systems biology, G-protein coupled receptors, mathematical modelling

## Abstract

To effectively navigate complex tissue microenvironments, immune cells sense molecular concentration gradients using G-protein coupled receptors. However, due to the complexity of receptor activity, and the multimodal nature of chemokine gradients *in vivo*, chemokine receptor activity *in situ* is poorly understood. To address this issue, we apply a modelling and simulation approach that permits analysis of the spatiotemporal dynamics of CXCR5 expression within an *in silico* B-follicle with single-cell resolution. Using this approach, we show that that *in silico* B-cell scanning is robust to changes in receptor numbers and changes in individual kinetic rates of receptor activity, but sensitive to global perturbations where multiple parameters are altered simultaneously. Through multi-objective optimization analysis we find that the rapid modulation of CXCR5 activity through receptor binding, desensitization and recycling is required for optimal antigen scanning rates. From these analyses we predict that chemokine receptor signaling dynamics regulate migration in complex tissue microenvironments to a greater extent than the total numbers of receptors on the cell surface.

## Introduction

Through interactions with non-hematopoietic stromal cells, B-cells generate tightly compartmentalized structures known as B-cell follicles within secondary lymphoid tissues. The follicular niche is responsible for coordinating B-cell homeostasis, activation and affinity maturation; precisely modulating B-cell activity through a temporal sequence of site-specific cues (1). The precise spatial positioning of B cells within the follicular niche is regulated by G-protein coupled receptors (GPCRs) that bind signaling lipids and chemokines inducing directed migration along a concentration gradient (2, 3). In this study we focus on CXCR5 the cognate receptor for CXCL13, a chemokine produced by stromal fibroblasts that is essential for follicle formation and maintenance (4–6). CXCR5 deficient B cells display aberrant migratory behaviors within lymph nodes, a phenotype associated with impaired homing to follicles (4, 7). CXCR5-/- B cells injected into a WT host are known to have different migration characteristics within LN follicles in comparison to WT B cells (8). Western blotting of pooled lymph nodes, *in vitro* migration assays, and measurements of the dissociation constant of CXCR5-CXCL13 suggest that the follicular concentration of CXCL13 ranges from 10-50nM. Studies of virally challenged lymph nodes however suggest that CXCR5-mediated migration can occur even when CXCL13 mRNA levels drop by 95% (8); this is consistent with studies that suggest a difference of 10 signaling receptors across a cell is sufficient to induce chemotaxis along a gradient (9, 10).

GPCRs are controlled by a complex and dynamic regulatory network that spans broad spatiotemporal scales, allowing cells to detect subtle asymmetries in chemokine concentrations (2, 10, 11). In terms of temporal sensing mechanisms, it has been reported that stable gradients of the homeostatic chemokines CCL19 and CXCL12 fail to promote the persistent directed migration of dendritic cells or neutrophils *in vitro*. Strikingly, rising chemokine concentrations were required to induce prolonged chemotaxis, in a mechanism dependent on GPCR kinase mediated negative regulation of receptor signaling (12). However, it is still unclear to what extent CXCR5 shapes gradient sensing within the primary B-follicle.

Despite recent advances in super-resolution imaging that permit single molecule analysis of GPCR dynamics *in vitro* (13), it is not yet possible to track CXCR5 dynamics *in situ*. To address this limitation many mathematical models have been developed to assess molecular dynamics through simulation analyses. The dynamics of CXCR4 and CXCR5 expression within the germinal center (GC) have been assessed using ordinary differential equations, an approach that predicts changes to components over one independent variable (e.g. time) on a continuous scale (14). Analysis of this model highlighted the importance of receptor down-regulation at the cell surface as a cell-intrinsic mechanism to govern migration patterns, predicting an oscillatory pattern of CXCR5 expression as B-cells migrated between the light and dark zones of the germinal center. Agent-based modeling, a technique that focuses on modeling the aggregate behaviors of individual cells or “agents” has also been employed to study this phenomenon. An ABM approach has been used to demonstrate that receptor internalization is a source of instability during tissue formation, leading to structural changes in tissue architecture (15) and that chemokine sensitivity is quickly down-regulated within the GC (16). Based on these experimental and theoretical studies, we hypothesize that receptor dynamics contribute to B-cell homeostasis by temporally regulating gradient sensing. However, much of the theoretical work that has been performed to date assume idealized chemokine gradients, and did not consider the complexity of tissue architecture, or the multimodal nature of CXCL13 diffusion (17). It is thus unclear if these models can accurately describe CXCR5 dynamics *in situ*.

To address our hypothesis that receptor dynamics contribute to B-cell homeostasis by temporally regulating gradient sensing we use the CXCL13Sim multiscale modeling platform (17) to assess the sensitivity of B cell scanning to perturbations in CXCR5 *in silico.* In previous work we have used CXCL13Sim to map the spatial distribution and dynamics of CXCL13 within lymph nodes. In this work we use the CXCL13Sim platform to ask how receptor dynamics regulate cell migration within complex tissue microenvironments. This novel system builds upon previous theoretical work on receptor dynamics by incorporating the 3-dimensional geometry of CXCL13^+^ follicular stromal networks, and the multimodal mobility of CXCL13 within the follicle.

Specifically, we apply parameter robustness to demonstrate that *in silico* B-cell scanning is robust to changes in receptor numbers and changes in individual kinetic rates of receptor activity, but sensitive to global perturbations where multiple parameters are altered simultaneously. This result is corroborated by a multi-objective optimization analysis to identify the configuration of CXCR5 signaling that maximizes the rate of antigen scanning. This analysis showed that for optimal scanning rates to occur multiple parameters regulating the dynamics of CXCR5 expression and signaling need to be tightly modulated. In addition, we perform single cell tracking to determine spatial and temporal patterns of expression in response to the complex gradients that occur *in vivo*. Taken in concert, our results suggest that the rapid modulation of chemokine receptor activity permits efficient antigen scanning by B-cells. This result has an important consequence for our understanding of B-cell migration in the context of complex tissue microenvironments which may benefit from targeting receptor dynamics, as opposed to downregulating total numbers of receptors.

## Materials And Methods

### Overview of the Development Framework

To ensure a principled and transparent design process we employ the CoSMoS (Complex System Modelling and Simulation) process, a framework to guide the modelling and analysis of complex systems (18). The design and implementation decisions made when constructing a simulator are influenced by the overarching scientific objectives of the work, with simulation results interpreted in this context (19, 20). To argue that the simulator fulfils its remit, acceptance tests, key design decisions, and information used to inform the design, development and validation of the model and simulation are presented as arguments over evidence using a visual notation derived from goal structuring notation (19). The argumentation structures are provided in files S3-S4 and can be opened using the ARTOO tool (http://artoofree.simomics.com/).

### Software Development and Computer Infrastructure

Within the simulation platform each module was developed using Java and the MASON^1^ ABM library version 19 (21) in an iterative process of implementation, validation and refactoring using Acceptance Test-Driven Development (ATDD) (22). Tests are continually assessed and refined as the project progresses and are incorporated into an automated regression framework using the java library JUnit^2^ with test coverage quantified using the eclipse plugin eclEmma^3^. Simulations were executed on a Linux Cluster made up of 64 CPU 256GB memory nodes and running Fedora 22 and an Oracle grid engine.

### Model Parameters, Parameter Ranges, and Outputs

#### Quantification of pLN Follicle Size and Cellularity

Follicle volume was obtained from two publications (23, 24). Kumar et al. determined the mean volume of a popliteal LN follicle using OPT scanning on BABB-immersed pLNs in conjunction with software based 3D reconstruction and quantification. Their analysis shows that typical pLN volume is 1.25mm^3^, dividing that by 10% (the percentage of volume accounted for by follicles) gives a volume of 1.25 x 10^8^ μm^3^ for all of the B- zones in one LN and dividing that by the number of follicles (18 [7-35]) gives 6.94 x 10^6^ μm^3^. In addition the authors observed a strong correlation (r^2^ = 0.93) between the size of the lymph node and the number of follicles. Values obtained by Irla et al. of inguinal lymph nodes suggest that total LN volume is 2.4mm^3^. Dividing this by 18% gives the % follicular volume. We then divide this value by the number of follicles (12.5) to get the volume of one follicle (3.46 x 10^7^ μm^3^). The discrepancy between the two sets of measurements can most likely be attributed to differences in lymph node morphology at different anatomical sites and different experimental procedures.

B-cell density was determined by quantifying CD19^+^ cells using flow cytometry in conjunction with Accucheck counting beads (n = 5 mice, 3 separate experiments) and averaging total pLN counts by the mean number of follicles per lymph node. Stromal cell densities within a fixed follicular volume were determined from the number of nodes obtained from a 400μm x 400μm x 30μm image (**Table 1**) of a follicle obtained from a *Cxcl13*-EYFP reporter mouse.

**Table 1:** Summary of model outputs.

Model Output	Description
**Total Displacement**	Record the steps taken by cells and calculate displacement over a fixed time period using vector addition.
**Net Displacement**	Euclidean distance between the first and last position of the cell
**Cell Velocity**	Total displacement/time
**Motility Coefficient**	Net displacement^2/^6* time
**Meandering Index**	√Time * (net displacement/total displacement)
**Scanning Rate**	Number of unique gridspaces reached within a single simulation run
**CXCR5 state**	A vector containing the number of free, ligand-bound, desensitized, and internalized CXCR5 molecules per cell, per timepoint

Following each individual simulation run, the following metrics are calculated for each B-cell agent.

From this data we define a popliteal lymph node as having a volume of 1.25 mm^3^, 15% of which is defined as a B-zone split between 15 follicles. Within this lymph node each follicle is spheroidal with a total volume of 1.25 x 10^7^ μm^3^ (~ 250 x 250 x 350 μm) and contains 4.8 x 10^4^ CD19^+^ B cells.

#### B-Cell Migration

The migration patterns of B cells were measured by injecting 5 x 10^6^ purified B cells from C57BL/6 or CXCR5 deficient mice labelled with the fluorescent cell staining dyes (((4-chloromethyl)benzoyl)amino)tetramethylrhodamine) CMTMR into age- and sex- matched wild type mice and imaged with two-photon microscopy (8). This data was provided by the Stein lab. WT B cells have a velocity (expressed as median[lower quartile – upper quartile]) of 8.0 [2.3 - 10.3] μm^2^min^-1^, a meandering index of 1.1 [0.1-2.4] and a motility coefficient of 15.6 [0.1 – 96.7] μm^2^min^-1^.

#### CXCR5 Expression

The number of CXCR5 molecules per naïve B-cells in lymph nodes is unknown, however data from similar systems suggests that the amount of receptors is in the range of 10,000 – 100,000 receptors (31, 32).

#### CXCL13 Diffusion Constant

A value of 7.6 ± 1.0 μm^2^s^-1^ was obtained using high-speed single-molecule imaging. An upper bound for this value was determined using the Einstein stokes relation, assuming a Stokes radius of 3.48 nm and that the molecule undergoes free diffusion in water (146 μm^2^s^-1^).

#### Kd and CXCL13 Secretion Rate

The total amount of chemokine from pooled lymph nodes (33), *in vitro* migration assays (5), and ligand binding constants (34) were used to derive upper and lower limits of a likelihood distribution for CXCL13 concentrations *in vivo*. Taken in concert these analyses suggest that the value lies in the range 1 - 50nM with a baseline follicle concentration assumed to be 10nM that we set as our binding affinity Kd. This is consistent with expected ranges of Kd for CCR7 and CCL19 where similar concentrations have been described to sufficiently trigger downstream signalling of CCR7 following binding of CCL19 in G-protein loading assays (35), downstream signalling assays (36) and microfluidic migration assays (37).

#### Ki and Kr

The two rate constants Ki and Kr associated with receptor internalization and recycling were estimated from experimental data on receptor desensitization and resensitization neutrophils and from mathematical modelling of B-cell migration (31, 32).

#### CXCL13 Decay Rate

Chemokine’s are further regulated *in vivo* by proteases providing rapid enzymatic modulation of bioactivity and availability. Systematic analyses of proteomic half-lives suggests a broad range of possible values, which we constrain between 15 minutes to 48 hours (38, 39). Assuming a constant decay rate this yields rates between 0.015 and 0.0002s^-1^ (40).

#### Calibration to Establish Baseline Simulation Behaviors

To constrain each parameter value, lower and upper bounds were set on the basis of direct experimental measures or derived from indirect evidence from the wider literature. Parameters were systematically changed and compared to experimental datasets using the non-parametric Mann-Whitney U-Test given outputs were not normally distributed, as determined by a Shapiro-Wilk test for normality (41). To assess the robustness of our baseline calibrated parameter values, outputs from best-fit parameter sets were compared to gene knockout experiments. Parameter sets that failed to reproduce statistically comparable results to both wild type and gene deficient mice were omitted from any further analyses.

#### Quantification of Model Uncertainty

To quantify sources of uncertainty in the our simulator we used the R software package SPARTAN (42). This package contains a suite of statistical techniques (described in more detail in the following sections) specifically designed to help understand the relationship between the simulator and the physical system it describes.

#### Quantification of Aleatory Uncertainty

CXCL13Sim is non-deterministic and therefore can produce different outputs under the same parameter inputs. To determine how many runs are required to give a representative output for a given parameter set we perform an aleatory analysis. To quantify aleatory uncertainty, 20 distributions were generated and contrasted for each sample size. A distribution of median responses for each simulation run is generated for each of the 20 subsets. Distributions 2–20 are contrasted with the distribution from the 1st set using the Vargha-Delaney A-Test (43), a non-parametric effect magnitude test that establishes scientific significance by contrasting two populations of samples and returning the probability that a randomly selected sample from one population will be larger than a randomly selected sample from the other. Values of 0.5 indicate that the medians are the same while values of 1 and 0 mean that there is no overlap. In our analyses we set thresholds for small (0.56), medium (0.66) and large (0.71) effect sizes based on values suggested by (44, 45) and define a significant behavioral alteration as one where the A-test statistic exceeds the medium threshold.

#### Local and Global Parameter Robustness Analyses

To quantify how sensitive simulation outputs are to perturbations in parameters we applied a number of sensitivity analysis (SA) techniques. For local analyses each parameter is adjusted, with all other parameters remaining at their calibrated value. The Vargha-Delaney A-Test described previously implemented in SPARTAN is employed to determine if changing the parameter value has led to a significant behavioral alteration in contrast to the baseline simulation. We define a significant behavioral alteration as one that surpasses a threshold A-test value of 0.66

To perform a global sensitivity analysis, we use two parameter sampling techniques, LHC (Latin-Hypercube) and eFAST (Extended Fourier Amplitude Sampling Test). Through Latin hypercube sampling, values for each parameter are selected with the aim of ensuring efficient coverage of the parameter space. Parameters that have significant impact on simulation behaviors are identified through calculation of a Partial Rank Correlation Coefficient (PRCC), a robust measure for quantifying non-linear relationships between an input and output (46). To calculate the PRCC the data are rank-transformed, and for each parameter, two linear regression models are found, the first representing the input parameter in terms of the other parameters and the second represents the output measures in terms of the other parameters. A Pearson correlation coefficient for the residuals from those two regression models gives the PRCC value for that specific parameter. Thus, PRCCs characterize a linear relationship between input x and output y after the linear effects of the other inputs on y have been discounted. A significance test is performed to assess if a PRCC is significantly different from zero (46).

Latin Hypercube Sampling (LHS) and Partial Rank Correlation Coefficients (PRCC) identify parameters that have a significant effect on model outputs. This approach facilitates an understanding of what parameters should be targeted to achieve a desired response but does not indicate which parameter uncertainties have the greatest impact on output variability. The extended Fourier Amplitude Sampling Test (eFAST) is a variance decomposition method that can be used to address this issue (47, 48). In this approach input parameters are varied, causing variation in model output. The algorithm then partitions the output variance, determining what fraction of the variance can be explained by variation in each input parameter. In this scheme, parameters values are sampled using a sinusoidal function of a particular frequency. Each parameter is taken in turn and sampled at a frequency that is much larger than the other parameters. Due to the symmetry of a sinusoidal function it is possible to choose the same parameter set more than once, therefore a re-sampling scheme in which a phase shift is introduced at each frequency is encouraged (46). Through Fourier analysis using these frequencies, variation in output can be partitioned between the parameters, giving an indication of the impact each has on simulation response. This process is repeated for an extra parameter, the ‘dummy’ parameter that has an arbitrary value range but no impact on simulation behavior. This enables a comparison between the impact of each parameter and one known to have no effect on simulation response. To quantify the influence of each parameter, two sensitivity indexes are calculated for each parameter-response pairing: a first-order (Si) and total order sensitivity (STi) index. The first indicates the fraction of output variance in that response that can be explained by the value assigned to the parameter. The latter indicates the variance in that response caused by higher-order non-linear effects between the parameter and the others under investigation. To determine the significance of these metrics, indexes are compared to those obtained for the ‘dummy’ parameter using a two-sample t-test.

#### Simulation Emulation With Machine Learning

The training dataset for emulator development was obtained using Latin hypercube sampling, with 1000 parameter sets. Each set was executed 100 times to mitigate aleatory uncertainty, and median responses calculated to summarize simulator performance under those conditions. The data set was partitioned into training (75%), testing (15%) and validation (10%) datasets.

The supervised learning approach used to generate CXCL13emulator was an artificial neural network, an approach inspired by the neuronal circuits in the brain, with computations structured in terms of an interconnected group of artificial neurons. During the learning phase, the weighting of connections between neurons are adjusted in such a way that the network can convert a set of inputs (simulation parameters) into a set of desired outputs (simulation responses). The ANN-based emulator was developed in the R package SPARTAN with supervised learning of the data achieved through backpropagation of errors. To determine optimal hyperparameters of the network we performed ten-fold cross validation on a selection of structures with thirteen inputs (the parameters) and four outputs (speed, meandering index, motility coefficient and checkpoints reached), with one to four hidden layers. The accuracy of each fold was determined to be the mean squared error between the predicted cell behavior responses and those obtained by the simulator, and the accuracy of the network structure determined to be the average of these ten-fold root mean squared errors.

#### Multi-Objective Optimization

Multi-objective optimization of the CXCL13emulator was performed using the non-dominated sorting genetic algorithm II (NSGA-II), a multi-objective genetic algorithm (49). This analysis was performed in R using the package mco v15.0^4^. The four objectives to be assessed by the algorithm were to: minimize the root mean squared error between emulator and simulator responses for cell speed, meandering index and motility coefficient; and maximize scanning rates. Values for generation number, mutation and crossover probabilities were determined by a global sensitivity analysis whereby values for mutation and crossover rates were sampled between 0.1 and 1.0 (intervals of 0.1) and values for the number of generations was sampled between 200 and 500 (intervals of 100). We chose parameters that performed well on all three objectives and maximized the variance of the parameter inputs.

## Results

**Figure 1:**
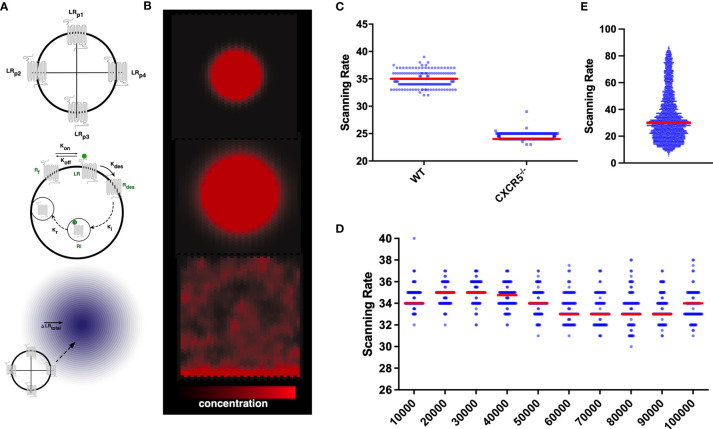
*In silico* CXCR5 modelling: Graphical Overview of the gradient sensing algorithm **(A)**, including receptor internalization and recycling **(B)** in the context of CXCL13 gradients **(C)** Quantification of scanning rates in wild-type and CXCR5 deficient B cells. **(D)** Quantifying scanning rates when perturbing total receptor numbers holding all other parameters fixed at baseline values. Median values are shown with error bars representing the I.Q.R. **(E)** Distributions of scanning rates observed from our global parameter perturbation analysis.

CXCL13Sim is a 3D hybrid multiscale model developed using the CoSMoS (Complex System Modelling and Simulation) process, a framework to guide the modelling and analysis of complex systems. In this scheme B cells are modelled as agents that adjust their behaviors with respect to vector and ordinary differential equation- based calculations adapted from a published scheme (50) that explicitly accounts for gradient detection and the dynamics of GPCR expression on the cell surface (**Figures 1A, B**). B-cell agents respond to CXCL13 gradients generated by *in silico* stromal cell networks. *In silico* stroma are modeled as a graph of nodes and edges (Module 1) and secrete CXCL13, which is represented by a double precision floating point number on a discretized grid with diffusion modeled using a discretized partial differential equation. A full overview of model parameters and outputs is provided in **Tables 1** and **2**). Within this scheme we find that the migration characteristics of *in silico* B-cells are consistent with those obtained by multiphoton imaging of both wild-type and CXCR5*^-/-^* B cells (**Figure S1A**) and that the topological parameters of the CXCL13^+^ follicular reticular network are consistent with those measured *in vivo* (**Figures S1B, C**). A full description of the design, implementation, and validation of CXCL13Sim, along with parametric and aleatory uncertainty quantification analyses, is provided in file S2. In Files S3-S4 we provide an argumentation structure detailing all of the key assumptions of our model and links to the data that supports our assumptions.

**Table 2:** Summary of parameter values.

Parameter	Value	Unit	Range	Reference
**B Cell Size**	7	μm	Constant	(25)
**Total Number of B cells**	6000	cells	Constant	Measured
**Total Number of MRCs**	100	cells	Constant	Measured
**Total Number of FDCs**	~200	cells	Constant	Measured
**Total Number of BRCs**	~450	cells	Constant	Measured
**Proportion of Cognate Cells**	5	%	Constant	–
**Displacement constant**	7.4	μm min^-1^	[1-10]	Calibrated
**Signal threshold**	10	ΔLR	Constant	(9, 10)
**Maximum turn angle**	180	Degrees	Constant	(8)
**Total receptor number**	48,000	Receptors	[10,000-100,000]	(11)
**K_on_**	4.8 x 10^5^	M s^-1^	[1x10^5^-1x10^6^]	(26)
**K_i_**	0.0033	s^-1^	[0.001-0.01]	(11, 27)
**K_des_**	0.075	s^-1^	[0.01-0.1]	(11, 27)
**K_r_**	0.004	s^-1^	[0.001-0.01]	(11, 27)
**K_off_**	0.0048	s^-1^	[0.001-0.01]	(11, 27)
**FDC secretion rate**	0.18	fg min^-1^ cell^-1^	[0.1-0.5]	(5, 28)
**RC secretion rate**	0.18	fg min^-1^ cell^-1^	[0.1-0.5]	(5, 28)
**CXCL13 decay rate**	0.007	s^-1^	[0.0002-0.05]	(29, 30)
**CXCL13 diffusion rate**	7.6	μm^2^ s^-1^	[0-146]	Measured
**α1 (measure of cell persistence during chemotaxis where lower values indicate higher polarity)**	0.475	–	0-1	Calibrated
**α2 (measure of cell persistence during random migration where lower values indicate higher polarity)**	3.8	–	Constant	Calibrated

For each parameter the name, baseline value and range used for uncertainty and sensitivity analyses is provided. Parameter values were determined experimentally or in cases where no direct experimental value exists, upper and lower limits were derived from indirect evidence, baseline values were then determined by fitting the model to experimental datasets (calibration). The model was further validated against migration data from CXCR5^-/-^ B cells and parameters were removed where possible. The values for stromal cells are averaged over 250 runs with individual values varying to a small extent between runs due to stochastic network formation.

To quantify parametric uncertainty in our simulation platform we apply sensitivity analysis (SA) techniques, which broadly speaking can be split into two categories: local and global. Local analysis techniques examine how robust the simulation is to a perturbation of a single parameter value (herein referred to as a One At a Time (OAT) analysis). However, local SA techniques cannot reveal compound effects where one parameter’s influence is dependent on the value of another. Such effects may be elucidated using global analysis techniques that perturb multiple parameters simultaneously.

To assess the consequences of CXCR5 deficiency on the induction of humoral immune responses we quantified B-cell trafficking of CXCR5^-/-^ B cells *in silico* and find a significant reduction in scanning rates (the number of unique gridspaces reached by an *in-silico* B-cell within a single simulation run) compared to wild-type B cells (**Figure 1C**). While complete loss of the receptor yielded a strong phenotype, perturbations to total numbers of CXCR5 across an order of magnitude (between 10-100,000 receptors) led to modest changes in the baseline rate of network scanning (**Figure 1D**). The distribution of scanning rates in this OAT analysis (**Figure 1D**), were far narrower than the distributions of scanning rates observed when performing a global multiparametric perturbation analysis (**Figure 1E**). This yielded the hypothesis that the dynamics of CXCR5 signalling modulate antigen scanning to a greater extent than total receptor numbers.

**Figure 2:**
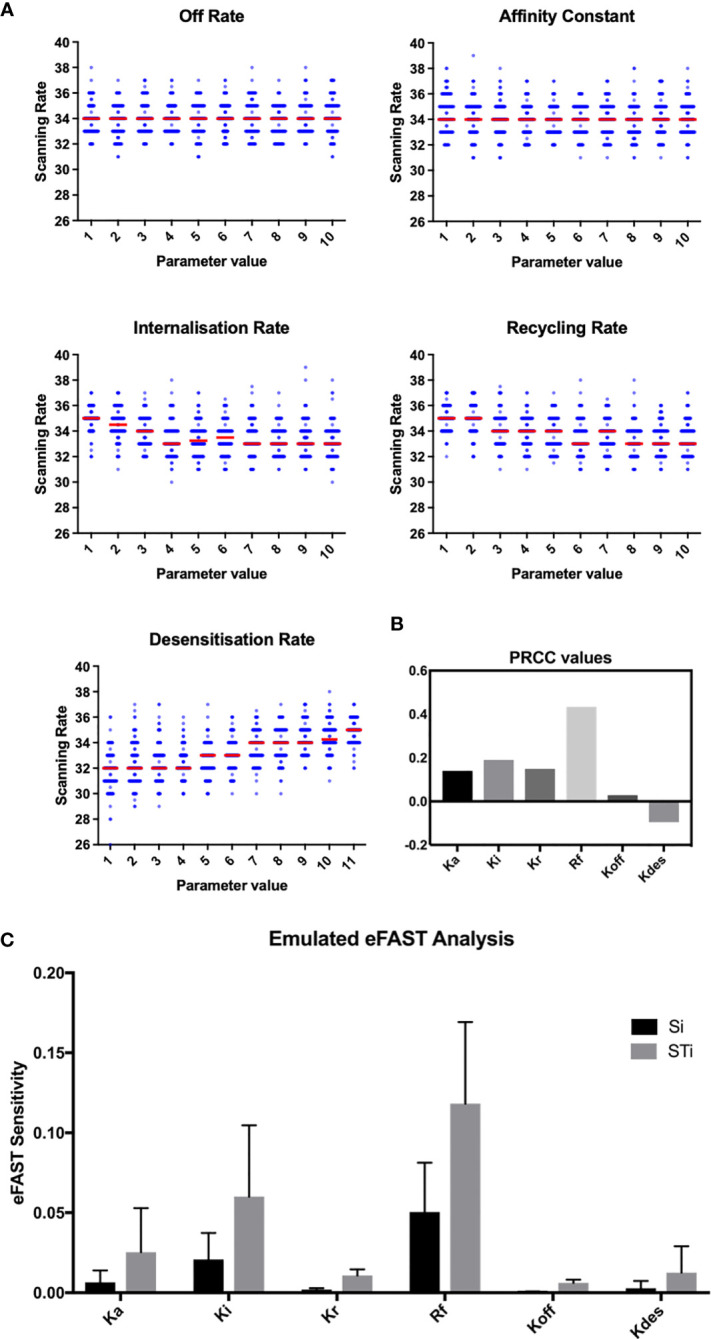
OAT perturbation of kinetic parameters that regulate CXCR5 signaling. **(A)** Perturbing kinetic parameters regulating CXCR5 activity and assessing the effect on *in silico* scanning rates. **(B)** Partial rank correlation coefficients quantifying the sensitivity of *in silico* scanning rates to perturbations in a given parameter, taking the influence of the other parameters into account. **(C)** eFAST analysis of parameter sensitivities using the CXCL13emulator Si (black) represents the fraction of output variance that can be explained by the value assigned to that parameter. STi (grey) represents the variance caused by higher order non-linear effects between that parameter and others explored. Bars represent the mean value for either Si or STi, with error bars representing the standard error over three resample curves.

**Figure 3:**
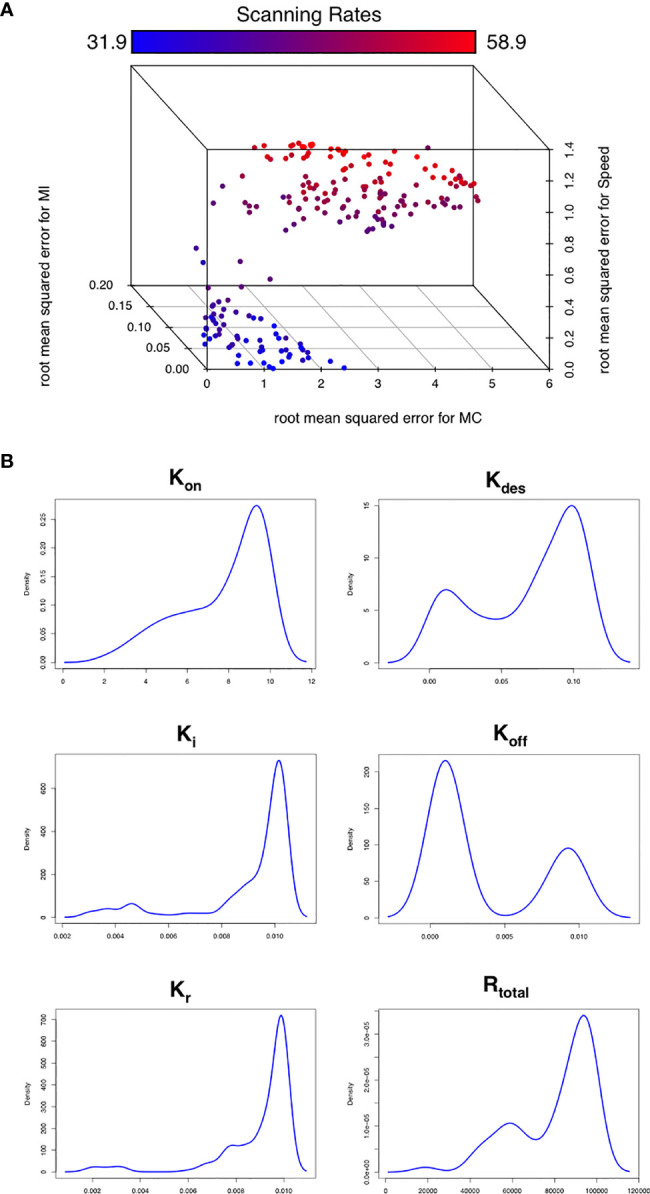
Optimizing CXCR5 signaling *in silico*. **(A)** Pareto front of solutions representing the optimal trade off in performance between different *in silico* migratory behaviors and scanning rates (color coded), using the multiobjective optimization algorithm NSGA-II. **(B)** Parameter distributions corresponding to the Pareto optimal solutions shown in **(A)**.

To assess this hypothesis we performed a suite of parameter sensitivity analyses, using the Spartan analysis software package in R (42). Using this approach, we find that overall scanning rates were robust to one-at-a-time perturbation to both K_on_ and K_off_ rates. This suggests that scanning is robust to the affinity of binding (defined as 1/K_d_ or K_on_/K_off._) over the ranges examined, and that efficient scanning can occur over a broad range of CXCL13 concentrations (**Figure 2A**). OAT perturbations to desensitization, K_i_ and K_r_ rates led to modest changes in scanning rates but global sensitivity analyses suggest complex interactions between these parameters, and that combinatorial perturbations may yield a synergistic effect (**Figures 2B, C**). Thus, using local and global sensitivity analyses we find that dynamic modulation of signaling has a greater impact on scanning rates than overall receptor numbers. To further explore this prediction, we perform multi-objective optimization (MOO) of the CXCL13emulator. To achieve this, we employ a specialized class of optimization algorithms known as multi-objective evolutionary algorithms (MOEAs) to determine what CXCR5 configurations maximize B-cell scanning rates. MOEAs use a similar principle to the germinal center (GC), using mutation and selection to determine a set of solutions (a Pareto front) where improvement in one objective cannot be obtained without compromising performance for another objective. In the GC this trade-off occurs because a mutation that improves binding for one epitope on a complex microbial antigen may reduce binding to other epitopes, reducing overall binding avidity. This GC-inspired analysis shows that our objectives are conflicting, with increased scanning rates leading to poorer agreement between emergent cell behaviors *in silico* and laboratory measures (**Figure 3A**). Analysis of the parameter distributions corresponding to the population of Pareto optimal solutions shows that R_total_,K_on_, K_des_, K_i_ and K_r_ are highly skewed towards high values while K_off_ is skewed towards lower values, consistent with the hypothesis that dynamic modulation of signaling through rapid receptor turnover and desensitization promotes effective migration (**Figure 3B**).

**Figure 4:**
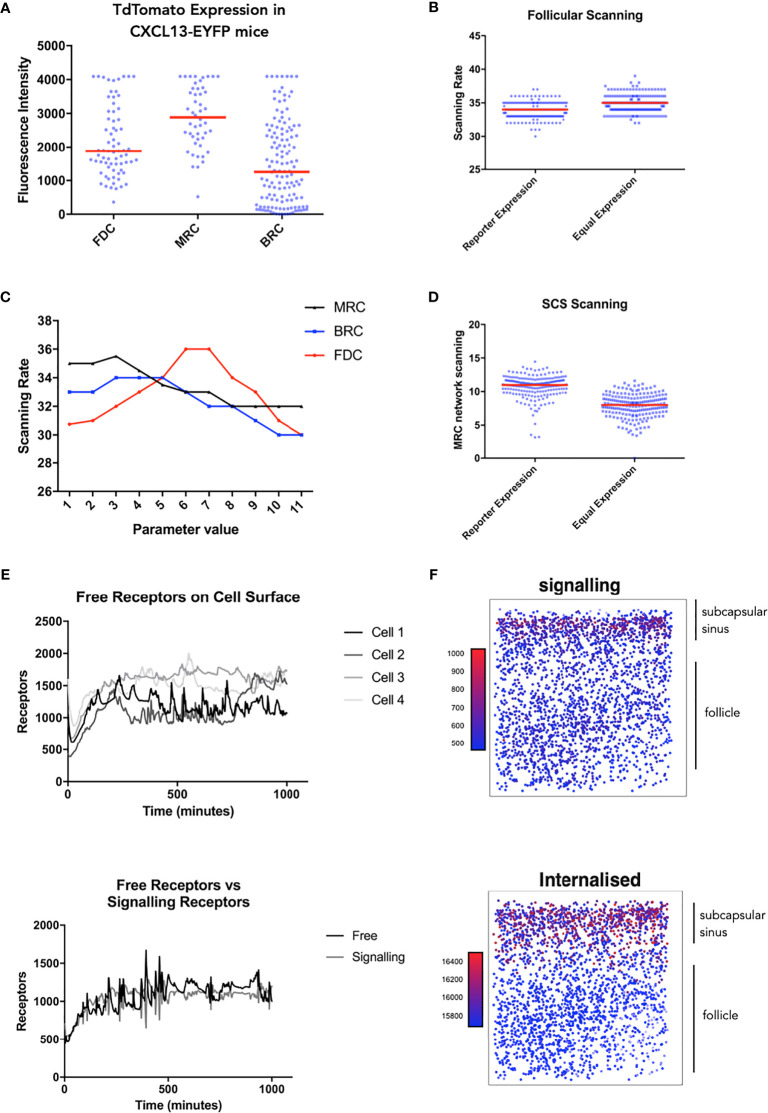
Single cell tracking *in silico* to assess the spatiotemporal dynamics of CXCR5 expression and signaling. **(A)** Experimentally obtained *in vivo* CXCL13 reporter expression from different stromal cell subsets in the CXCL13-EYFP mouse. **(B)** *In silico* scanning rates for the entire follicle using equal secretion rates for all stromal cell subsets or secretion rates representative of CXCL13 reporter expression. **(C)** *In silico* scanning rates following OAT perturbations to secretion rates in different stromal cell subsets. **(D)** *In silico* scanning rates for the subcapsular sinus using equal secretion rates for all stromal cell subsets or secretion rates representative of CXCL13 reporter expression. **(E)** Single-cell tracking of CXCR5 expression on the cell surface. (Top) each line represents a distinct B cell within the same simulation run and (bottom) comparison of free and receptor signaling dynamics within a single cell. **(F)** Spatial dependence of CXCR5 signaling within the follicle. Each dot in the diagram represents the X and Y coordinates of a B cell agent in the simulator. The top of each square diagram is the subcapsular sinus. Each agent is colored by the number of receptors (as indicated by the title of each plot) with red representing high values and blue representing low values.

Taken in concert, our data suggest that modulation of receptor activity is a key determinant of trafficking behaviors. Leveraging recent insights into CXCL13 gradient formation (17), we use simulation analysis to resolve spatial and temporal components of the CXCL13-CXCR5 regulatory axis. To assess cell specific contributions to follicular scanning rates we performed histological analysis of lymph node tissue sections from Cxcl13-Cre/Tdtomato R26R-EYFP (abbreviated as Cxcl13-EYFP) mice (51). In Cxcl13-EYFP mice, EYFP acts as a lineage marker, endogenously expressed in cells that originate from a CXCL13-producing precursor, while TdTomato expression (red fluorescent protein, RFP) is confined to cells with current CXCL13 promoter activity. Within the CXCL13^+^ stromal cell populations we define 3 subsets: CXCL13^+^ CD21/35^+^ Follicular Dendritic Cells (FDCs) and CXCL13^+^ CD21/35^−^ reticular cells (CD21^−^ RCs) comprising reticular cells located underneath the subcapsular sinus (marginal reticular cells - MRCs), and at the outer follicle (B-zone reticular cells - BRCs) (17). Using the relative TdTomato intensities as a proxy for CXCL13 secretion we infer distinct secretion rates for each stromal cell subset. Using this secretion profile, we find no significant change in overall scanning rates (**Figures 4A, B**) but did find that B-cells localize at the subcapsular sinus, a key site for antigen presentation (**Figure 4D**). To further assess how relative secretion rates of stromal cells can affect scanning rates we performed a sensitivity analysis and find that overall scanning rates are highest with low rates of secretion from reticular cells, while for FDCs highest rates were found at intermediate secretion rates (**Figure 4C**). We then followed the temporal (**Figure 4E**) and spatial (**Figure 4F**) dynamics of CXCR5 expression on individual B-cell agents. Analysis of signaling and free receptors on the cell surface shows a dynamic pattern of expression (**Figure 4E**). Analysis of each receptor subset shows that desensitized receptors were the highest CXCR5 subset followed by internalized receptors and relatively few free and signalling receptors on the cell surface (**Figure 4F**). Interestingly, CXCR5 expression was spatially regulated with highest levels of signalling occurring at the subcapsular sinus, a profile associated with low numbers of free receptors and high numbers of internalized receptors (**Figure 4F**).

## Discussion

CXCR5 is a key molecular player in CXCL13-mediated cross talk between B cells and stromal cells. *In vitro* studies of GPCR-mediated migration have highlighted a highly dynamic and intricate regulation network at the cell surface that allows immune cells to dynamically perceive the localized environment. Translating these findings *in vivo* is challenging due to the limitations of currently available experimental approaches. In this study we apply novel computational tools to address this limitation, and have explored the robustness of B cell scanning to perturbations in CXCR5 signaling and expression *in silico*.

Specifically, we extend upon previous models of chemotaxis *in vitro*, and *in vivo* models of lymph node stromal cells to better examine the migration of B cells within a complex tissue microenvironment. The use of ordinary differential equations within each agent permits single-cell analyses of CXCR5 expression with high spatiotemporal resolution. This key advantage allows the tool to serve as an adjunct to experimental approaches that are not capable of performing single cell analyses *in situ.* Flow cytometry approaches require cells to be isolated from a tissue, while confocal microscopy takes a static snapshot of a dynamic process. While single-cell sequencing is becoming more prevalent, linking this information back to spatial positioning within a tissue is challenging, with need for a robust panel of markers that differentiate cell subsets on the basis of spatial positioning or the use of micro-dissection. Similar issues arise when experimentally measuring CXCL13 within a tissue: fluorescent reporter systems cannot be used to tag the molecule itself, as the fluorescent label would drastically outweigh the molecule and alter its diffusive and binding characteristics. Our simulation platform may thus serve as a useful adjunct to further experimental studies of chemokine receptor activity, affording single-cell tracking precision within a 3-Dimensional complex environment beyond the capability of current imaging approaches.

Based on our quantification of parametric uncertainty, we find that while some kinetic parameters were unidentifiable, non-linear interactions between kinetic parameters were driving simulation outputs, a finding consistent with a meta-analysis of parametric uncertainty in systems biology models (52). Importantly, this result was observed across many different parameter combinations and model iterations. In future work we aim to leverage this insight to simplify our model such that it can be generalized to other contexts.

Focusing on CXCL13-CXCR5 mediated regulation we find that *in silico* scanning of the LN follicle was robust to changes in total receptor numbers while complete loss of the receptor yielded a significant phenotype. This result is consistent with experimental studies showing that CXCR5 migration is also important in virally infected lymph nodes, where *cxcl13* gene expression is significantly reduced (8). This result, in conjunction with a suite of local and global sensitivity analyses led to the hypothesis that the dynamic modulation of CXCR5 signalling through rapid turnover and desensitization of receptors is a key determinant of antigen scanning. To explore our prediction, we used an MOEA approach to determine what combination of CXCR5 associated parameters gave rise to the highest scanning rates. Consistent with our hypothesis, this analysis supports a model whereby dynamic turnover of receptors and modulation of signalling are critical. Parameter distributions that gave rise to Pareto optimal solutions were skewed towards high rates of receptor ligand-binding, internalization, recycling, and desensitization rates.

To place our results in the context of the tissue microenvironment we also analyzed spatial and temporal aspects of CXCR5 activity. We assessed cell specific contributions to follicular scanning rates and show that FDCs are a key determinant of scanning within the primary lymph node follicle. Interestingly, using the fluorescent intensity values for each cell types to inform relative secretion rates yielded a CXCL13 landscape which promoted scanning at the subcapsular sinus, a site where large antigen enters the LN. This configuration may also promote shuttling of antigen by naïve B cells to the FDC network for long-term storage. In addition, we performed single cell tracking experiments that predict that CXCR5 expression on the cell surface is dynamic, an emergent property that has been reported in other theoretical studies (14). Interestingly, CXCR5-mediated signalling was also spatially regulated – an emergent property where highest rates of signalling were observed at the SCS, close to the site of antigen entry into the lymph node parenchyma. Given our data, we hypothesize that dynamic modulation of receptors at the cell surface leads to fine-tuning of migratory responses within the B-cell niche. However, further analyses are required to determine the contribution of other chemotactic molecules and adhesion molecules to this phenomenon.

In further studies, we aim to extend this system by incorporating additional migratory factors such as CCL19/21, CXCL12 and 7,25α hydroxycholesterol (1). The decision to omit these factors from the model was influenced by the lack of quantitative data for these molecules rather than a limitation of the computational platform. By incorporating these factors as more data becomes available we can begin assessing complex interactions between different receptors.

To summarize, our computational approach has enabled us to study the dynamics of chemokine receptor expression in the context of a complex tissue microenvironment. This work builds upon previous theoretical studies which study receptor dynamics in the context of idealized morphogen gradients where the complexity of tissue architecture is highly abstracted. Our data suggest that while receptor numbers are important in regulating migration they influence scanning rates to a lesser extent than the combined effect of receptor binding, desensitization, and internalization. The data also suggests that CXCR5 activity is modulated differently within different subfollicular niches. As such, our simulation platform is well placed to complement experimental work, given the relative speed at which data is acquired, the ability to perform single cell analyses, and the ability to assess how perturbations at one level of organization manifest at different scales.

## Data Availability Statement

The datasets presented in this study can be found in online repositories. The names of the repository/repositories and accession number(s) can be found below: https://www.kennedy.ox.ac.uk/technologies/resources/cxcl13sim.

## Author Contributions

JC designed and performed the experiments, analyzed, and interpreted the data and wrote the manuscript. KA and JS performed experiments, analyzed data, provided key reagents, intellectual input, and technical assistance. JT and MC designed experiments, analyzed and interpreted results, coordinated the research, and wrote the paper. All authors contributed to the article and approved the submitted version.

## Funding

KA was supported by Wellcome Trust Centre for Future Health grant (204829), JT by EPSRC grant EP/K040820/1. JC, JT, and MC were funded by Wellcome Trust (Computational Approaches in Translational Science WT0905024MA, HFSP (RGP0006/2009 MC) and Medical Research Grants MR/K021125/1 and G0601156. MC is funded by the Kennedy Trust.

## Conflict of Interest

The authors declare that the research was conducted in the absence of any commercial or financial relationships that could be construed as a potential conflict of interest.

## Publisher’s Note

All claims expressed in this article are solely those of the authors and do not necessarily represent those of their affiliated organizations, or those of the publisher, the editors and the reviewers. Any product that may be evaluated in this article, or claim that may be made by its manufacturer, is not guaranteed or endorsed by the publisher.

## Footnotes

1 Available from http://cs.gmu.edu/~eclab/projects/mason.

2 Available from http://junit.org/junit4/.

3 Available from http://www.eclemma.org/download.html.

4 Available from https://CRAN.R-project.org/package=mco.
