# Supplementary material for: Simulating CXCR5 Dynamics in Complex Tissue Microenvironments
Source: Front Immunol. 2021 Sep 7;12:703088. doi: 10.3389/fimmu.2021.703088 (PMC8452942; doi:10.3389/fimmu.2021.703088)
Supplement: Supplementary file 1 [file DataSheet_1.pdf]

Supplementary Material

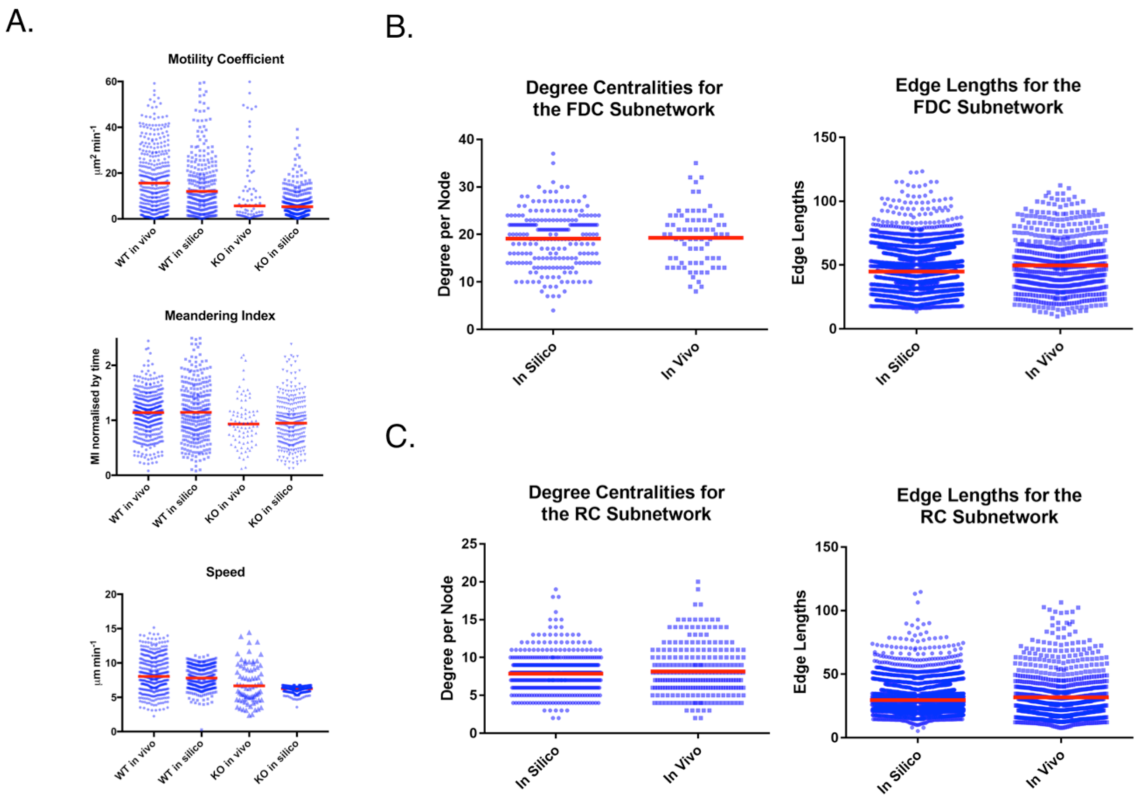

Supplementary Figure 1. (A) Comparison of in silico and in vivo migration patterns in B cells. (B-C) Comparison of in silico and in vivo network topology parameters.
