# Supplementary material for: Simulating CXCR5 Dynamics in Complex Tissue Microenvironments
Source: Front Immunol. 2021 Sep 7;12:703088. doi: 10.3389/fimmu.2021.703088 (PMC8452942; doi:10.3389/fimmu.2021.703088)
Supplement: Supplementary file 2 [file DataSheet_2.pdf]

Section 1. Overview of the Development Methodology

To ensure a principled and transparent design process we employ the CoSMoS (Complex System Modelling and Simulation) process, a framework to guide the modelling and analysis of complex systems (**Figure 1**) [1,2]. Initial stages of model design involve the development of a domain model, a non-executable, conceptual model focusing purely on current biological understanding, disregarding any consideration of how to implement and simulate the conceptual model. The domain model specifies the states, relationships and methods of interaction (the rule-set) for the biological entities being captured. The platform model is akin to a software specification, as is standard in software engineering, and details how the biological processes specified in the domain model are to be implemented. The simulation platform is an executable piece of software, which implements the underlying conceptual model. The results model provides a structure to interpret data obtained from the Simulation Platform. A specification is created that documents the output obtained from the simulation, what domain knowledge this is compared to, and the statistical methods used to assess this result.

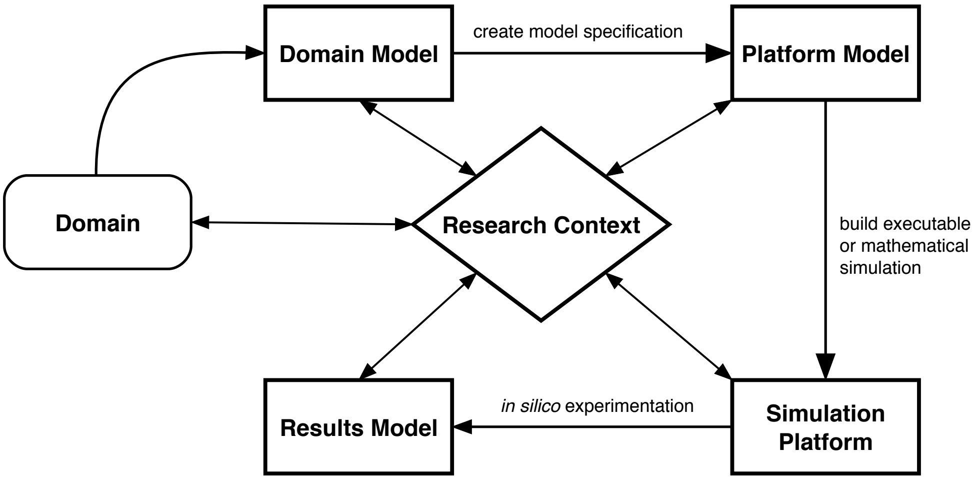

Figure 1. Overview of the CoSMoS process. In this approach the biological system of interest is referred to as the domain. Initial design phases lead to the development of a domain model, a non-executable specification focusing on current understanding with respect to the research context. The platform model represents a software blueprint while the simulation platform is an executable piece of software that implements the conceptual model. The results model summarizes the understanding generated from experimentation conducted using the simulator [1]

Section 2. Domain Model Development

In this section we detail the Domain Model. The research context of the simulator is summarised using an expected behaviours diagram. This diagram specifies the research question and the observed emergent properties of the system, as well as the biological entities and mechanisms hypothesised to give rise to these properties. This approach helps to scope the research context, highlighting key model entities and time points of interest.

Following this step low level behaviors are modelled using an adaptation of the Unified Modelling Language (UML) [3]. The Unified Modelling Language (UML) is a general-purpose visual notation used to model the design of a system and is commonly used in software engineering. Specifically, we use two key diagrams: (i) A **State Machine Diagram** where for each model entity, states (a set of attributes and behaviours associated with a model entity at a specific moment in time) that the entity can exist in and the interactions that much take place for the state to change are examined and documented (**Figure 2**). (ii) An **Activity Diagram** where we specify a sequence of activities associated with model entities. For each entity, it details the workflow from an initial state to a finish point, detailing the decision paths and interactions with other entities that occur (**Figure 3**).

To argue that the simulator fulfils appropriately addresses the research context, acceptance tests, key design decisions, and information used to inform the design, development and validation of the model and simulation are presented as arguments over evidence using a visual notation derived from goal structuring notation (GSN) and can be opened using the GSN visualisation tool ARTOO [4].

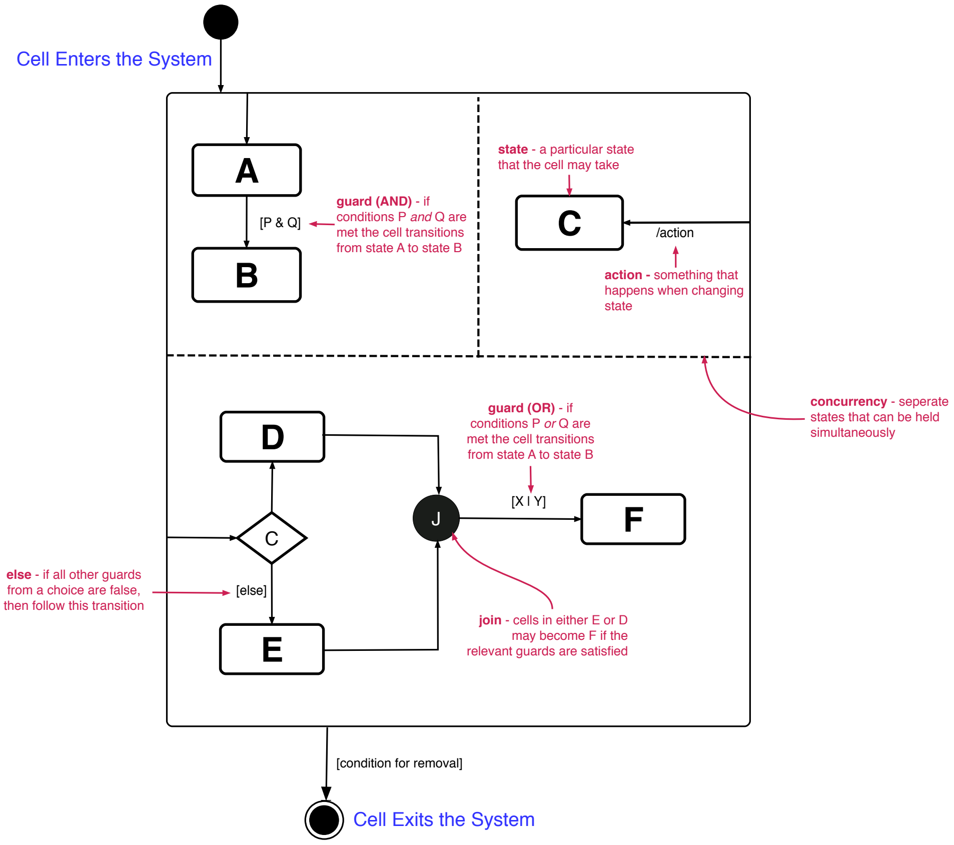

Figure 2. Key for Adapted Unified Modelling Language: State Machine. This is a key for the syntax used to describe biological processes as state machine diagrams in both the domain and platform models. *Rectangles* represent actions and agent states; *diamonds* represent decisions; *arrows* represent the order in which state transitions/activities may occur while a *circle* represents entry/exit into the system.

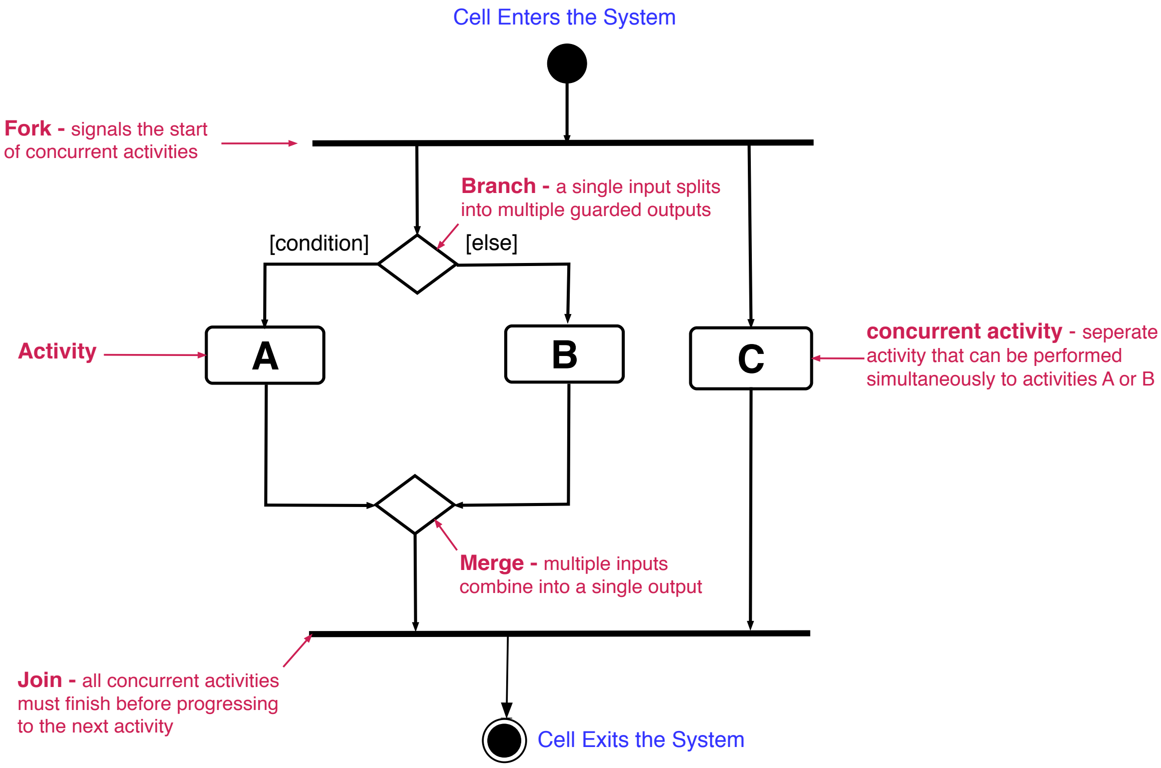

Figure 3. Key for Adapted Unified Modelling Language: Activity Diagram. This describes the syntax used to describe biological processes as activity diagrams in both the domain and platform models. *Rectangles* represent *actions or activities*; *diamonds* represent *decisions*; *horizontal bars* represent the start (*split*) or end (*join*) of concurrent activities; a *black circle* represents the start (*initial state*) of the workflow while an *encircled black circle* represents the end of the activity (*final state*).

2.1 Overview of the Biological System

Following immunization and infection, complement activation and deposition onto microbial antigens contributes to their efficient transport to the LN in a soluble form or through active transport by Dendritic Cells (DCs). Within the lymph node, antigen smaller than 70 kDa with a molecular radius less than ~4 nm filters into the parenchyma through reticular conduits or small pores in the subcapsular sinus [5]. The conduit system is guarded by PLVAP, a protein that forms a molecular sieve designed to regulate the parenchymal entry of lymphocytes and soluble antigens [6]. However, not all lymph-borne molecules have free access to the lymphocyte compartment; larger antigen is retained at the subcapsular sinus, where macrophages and DCs sample the lymph and remove microorganisms, larger molecules and debris [7].

Within the B-zone a dense web-like network of non-haematopoietic stromal cells are found. This network comprises 3 distinct but interconnected CXCL13^{+} cell types: Marginal Reticular Cells (MRCs), B-zone Reticular Cells (BRCs) and Follicular Dendritic Cells (FDCs). FDCs not only express CXCL13 but also act as an important reservoir for trapped antigen that can stimulate naïve B cells [8].

Concurrently, B cells migrate across the endothelium into the cortex through the HEV at a rate dependent on the local vasculature. The arrest of blood-borne B cells in HEVs requires binding of the chemokine receptors CXCR4, CCR7 and to a minor extent CXCR5, leading to activation of the adhesion molecule LFA-1 that binds to ICAM-1 and ICAM-2 [9]. Once inside the lymph node, access to the follicle requires the G-protein-coupled receptor (GPCR) CXCR5 and is promoted by ICAM-1-expressing fibroblastic reticular cells (FRC) of the T-cell area which act as guidance structures [9].

As B cells scan the follicle they respond to ligands for the receptors CXCR5 and EBI2; this promotes contact with FDCs and cells located around the follicle perimeter, including sinus associated macrophages, MRCs and DCs [10–13]. Non-cognate interactions with antigen via complement receptors CR1 and CR2 facilitate the mass transport of opsonized antigens from the exposed follicle perimeter to the protected centre for long-term display on FDCs, where they are trapped in the form of

iccosomes by complement receptors present on the dense network of FDC processes [8].

If lymphocytes fail to recognize cognate antigen within a few hours to days, they return to the circulation in a sphingosine-1 phosphate receptor 1 (S1PR1) dependent manner through efferent lymph vessels and the thoracic duct [14,15]. Notably, naïve B cells are resident in the LN longer than either $CD4^+$ or $CD8^+$ T cells [16]. However, if a B-cell does meet its cognate antigen it can internalise the antigen through its B-cell receptor. It can then degrade pathogen proteins into peptides for display on Major Histocompatibility Complex Class II (MHC-II) molecules on the B-cell surface. Following antigen priming, a B cell upregulates CD86 and CD80, proteins that provide co-stimulatory signals for T-cell activation while upregulation of the lymphocyte activation antigen CD69 inhibits the egress activity of S1PR1, leading to retention in the lymph node [12]. To maximise encounters with T cells, antigen-primed B cells exhibit a reduction in migration velocity, upregulate CCR7, (while CXCR5 expression remains unchanged) distribute themselves along the B/T border as a result of the balanced chemoattraction of CCL19/CCL21, CXCL13 and 7α,25 hydroxycholesterol [12].

Helper T cells, primed by dendritic cells earlier in the infection, migrate around the T-zone in a CCR7 dependent manner. A subset of activated $CD4^+$ T cells upregulate CXCR5 and reduce CCR7 expression allowing them to migrate towards the edges of follicles to provide help to B cells. This activation occurs via CD40 ligation subsequently driving the resting B-cell into the cell-cycle and upregulating the transcription factor bcl6, therefore reducing the propensity to undergo apoptosis [17]. Activated B cells migrate to interfollicular and outer follicle regions after receiving T cell help, where they can undergo proliferation for 1-2 days before returning to the centre of the follicle in an S1PR2 dependent process to initiate GC clustering and acquire a GC phenotype associated with, amongst other molecules, the upregulation of the chemokine receptor CXCR4 and the glycan moiety GL7 [18,19]. If BCs fail to acquire this TC help they revert back to a naïve phenotype, downregulating CCR7 and CD86 [20].

2.2 Characterising Current Understanding Through Visual Notations

The aim of this Domain Model is to summarise current understanding of the pathway with respect to the following question: *How does CXCL13 regulate the positioning of cognate B cells within primary lymph node follicles?* We define the research context using an expected behaviours diagram (**Figure 4**), and detail the behaviours of model entities using state-machine and activity diagrams (Figures 5-9) [3].

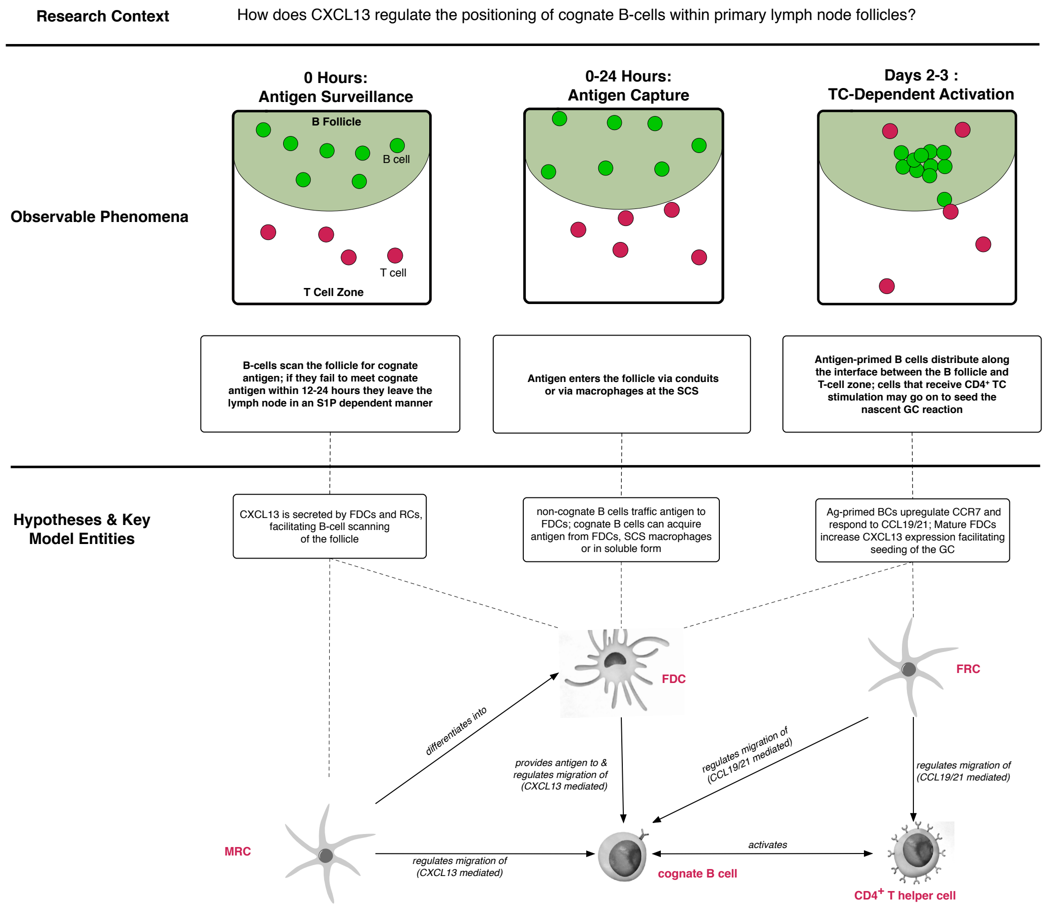

Figure 4. Expected Behaviours Diagram for CXCL13Sim. In this diagram we present the research context within which the model is developed. This diagram specifies the key emergent properties of the system as well as the low level mechanisms reported to give rise to these properties. Lastly, it defines key entities in the model and the relationships between them.

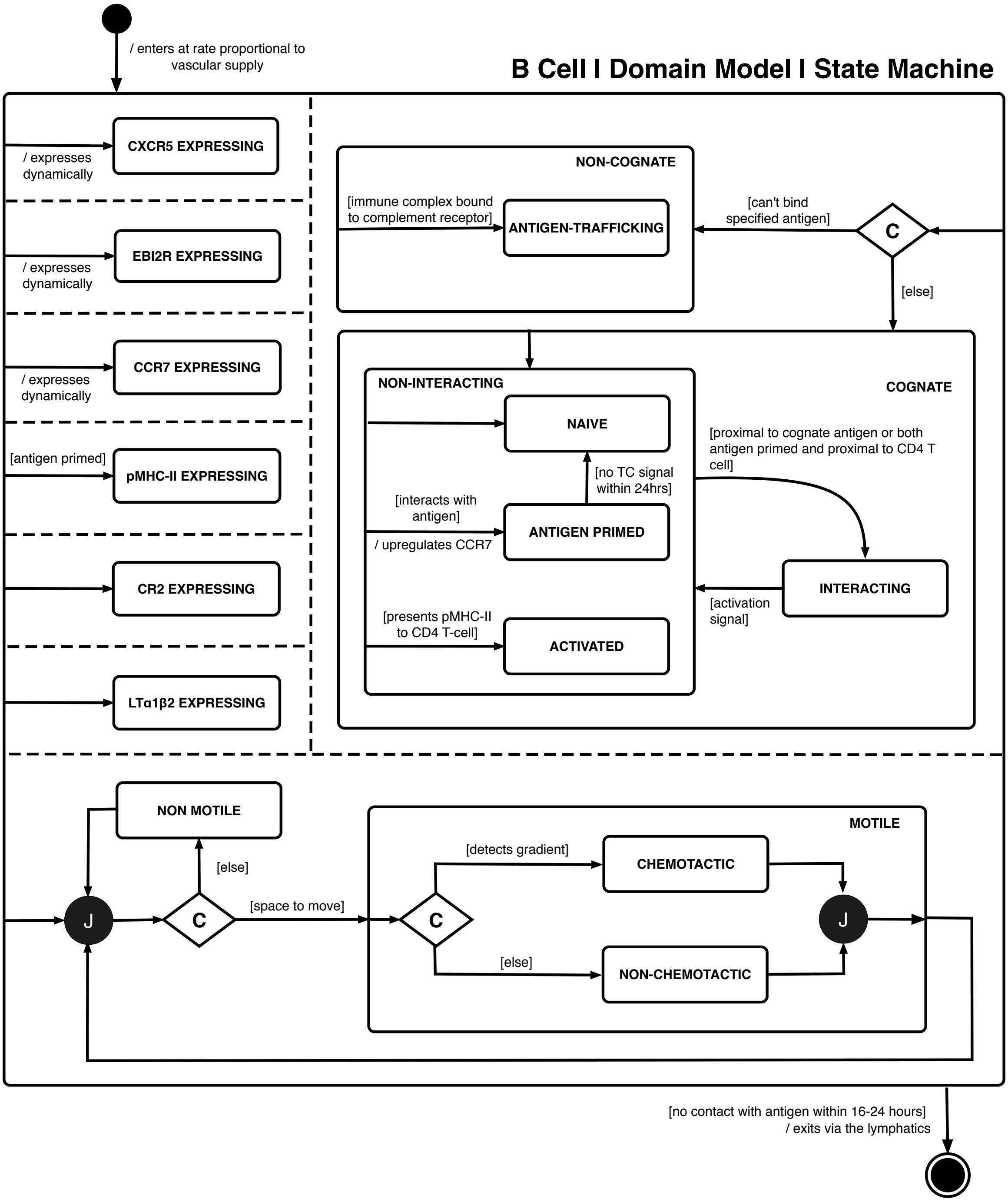

Figure 5. State Machine Diagram for a B cell: A B cell enters the LN at a fixed rate dependent on vascular supply, once in the LN a cognate B cell can become MHC expressing if it encounters antigen. Each B cell dynamically expresses CXCR5 and EBI2R and through detection of chemokine gradients can decide to move chemotactically or randomly if there is sufficient space to move. B-cells continue to express LTα1β2 at a fixed level and if 12-24 hours have passed and no interactions have occurred, the B cell exits the LN. If however, a BC encounters antigen it becomes antigen-primed, upregulating pMHC-II and CCR7 to facilitate interactions with T cells.

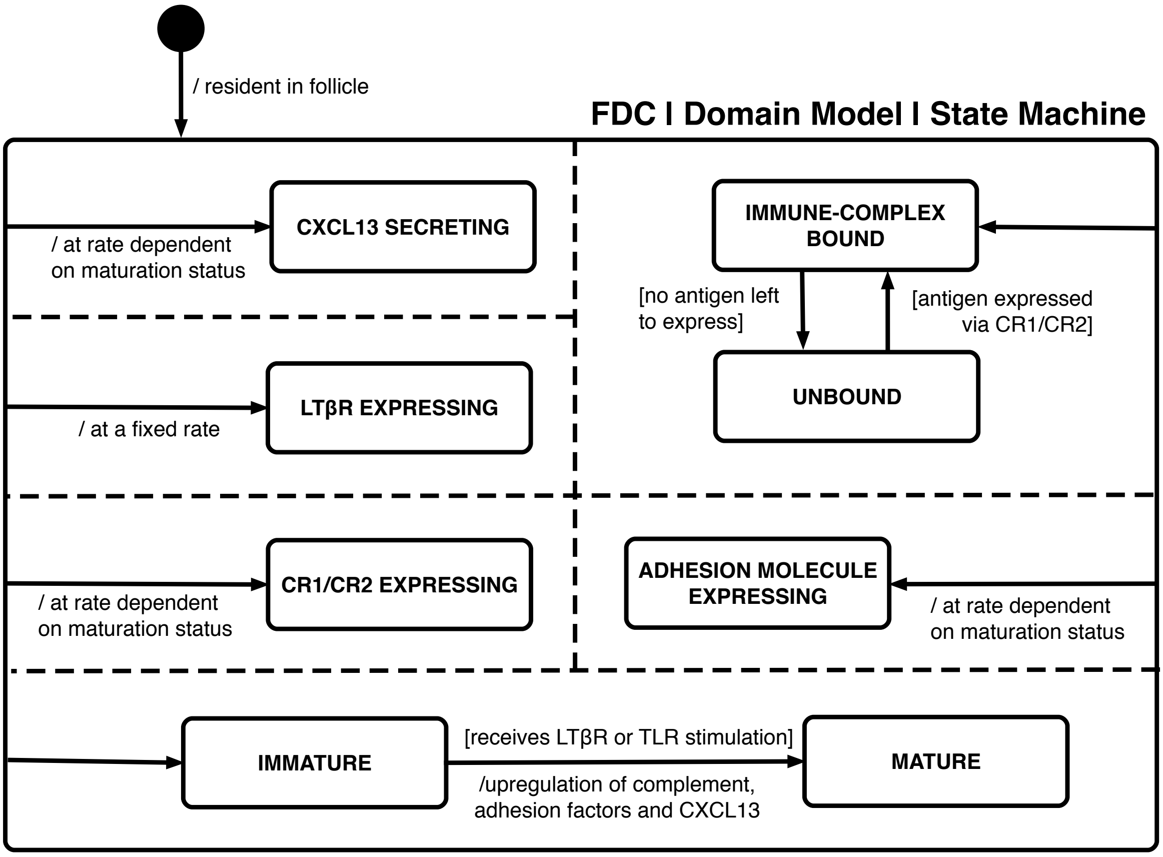

Figure 6. State Machine Diagram for a FDC: FDCs are resident in the system and are antigen presenting at time zero. FDCs secrete chemokine and express LTβR, complement receptors and adhesion molecules at a rate dependent on maturation status. FDCs also retain and present antigen unless cognate B cells capture all antigen

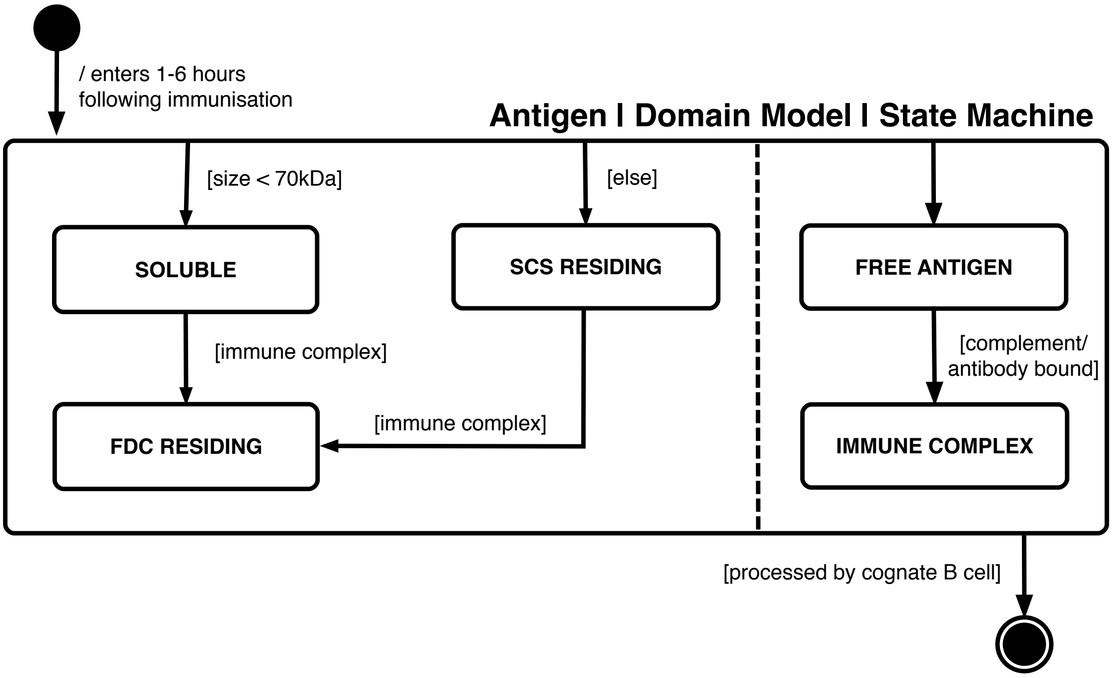

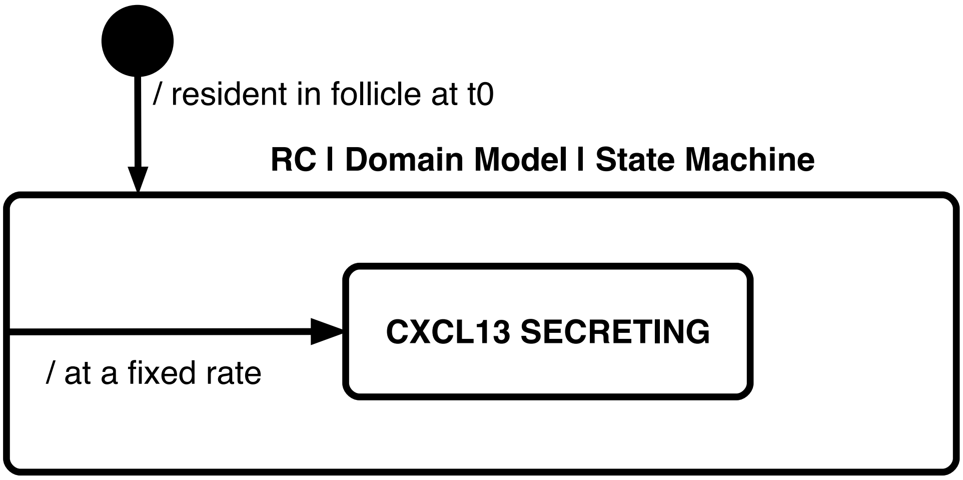

Figure 7. State machine diagrams for antigen and RCs: Antigen enters the follicle 1-6 hours following immunisation. It enters in a soluble or SCS residing form but may be transferred to FDCs if part of an immune complex. RCs are resident in the follicle at time zero and secrete CXCL13 at a fixed rate. SCS macrophages are in the system at time zero and can present antigen.

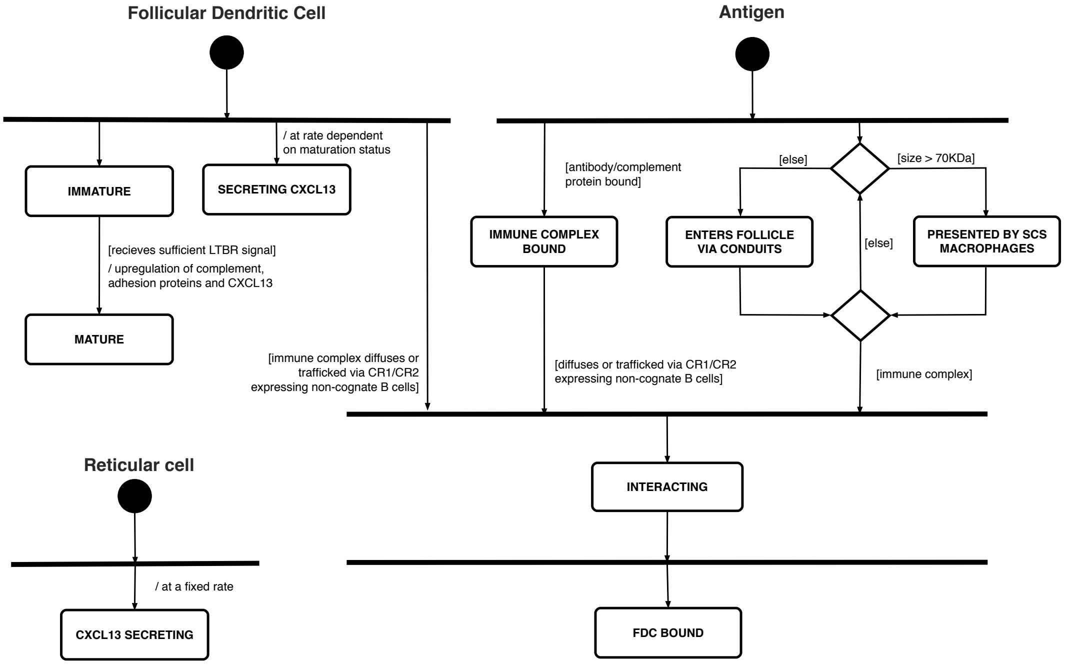

Figure 8. Activity Diagram 1: These diagrams detail the activities performed by each model entity and the conditions required for a change of activity to occur. At the beginning of the activity FDCs exist in an immature state but mature if sufficient LTβR signal is received. CXCL13 is secreted at a rate dependent on maturation status. Antigen enters the system via the conduits or may be presented by SCS macrophages if greater than 70kDa. If antigen is part of an immune complex it may become FDC bound. RCs secrete CXCL13 at a fixed rate.

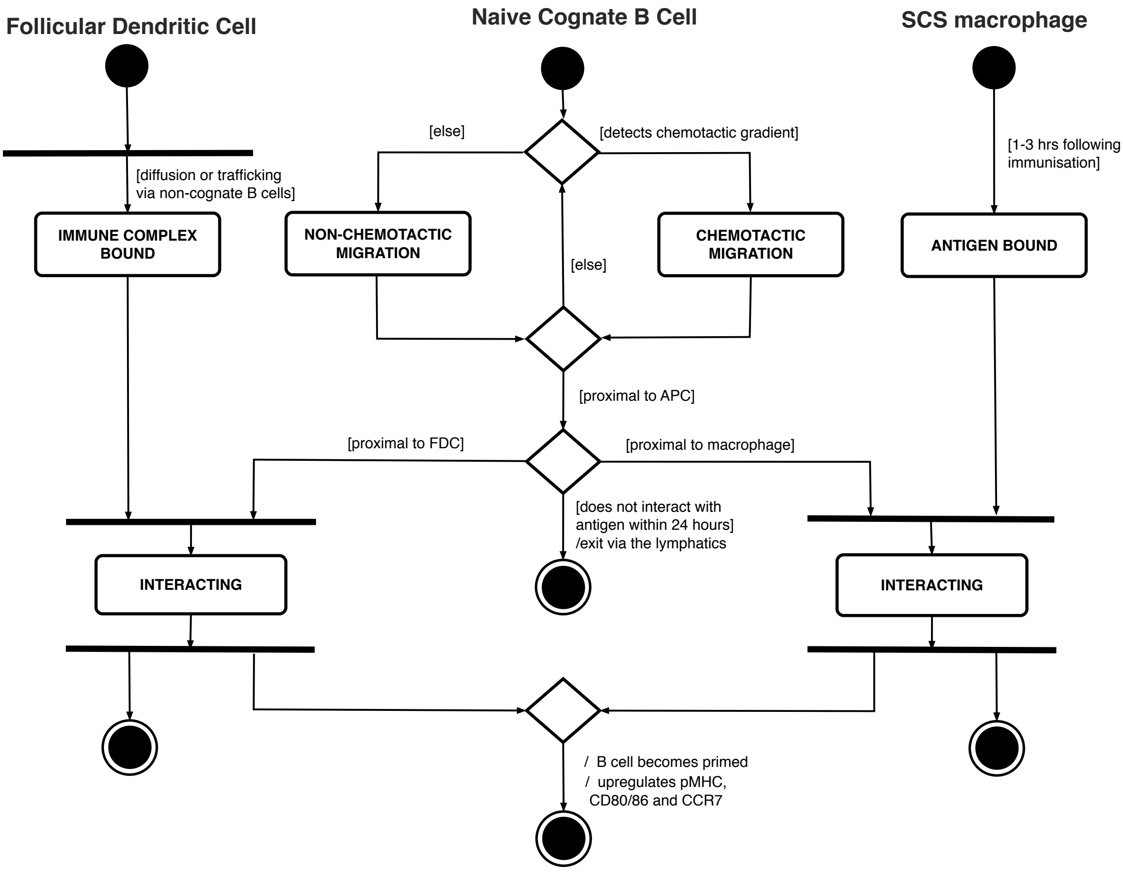

Figure 9. Activity Diagram 2: Activity diagrams detail the activities performed by each model entity and the conditions required for a change of activity to occur. At the beginning of the activity FDCs express antigen unless cognate B cells capture all antigen. Under homeostatic conditions FDCs secrete CXCL13 at a fixed rate. If a B cell expresses CXCR5 then it can orientate itself along a CXCL13 gradient, otherwise it randomly orientates itself and if there is free space available can migrate. Once proximal to an FDC/SCS macrophage a cognate B cell may interact with antigen via its B-cell receptor and express it via MHC-II.

Section 3. Platform Model

The Domain Model detailed in **Section 2** describes a set of biological processes occurring on molecular, cellular and tissue levels of organization. To provide a fit for purpose representation of the domain model while also promoting model parsimony and efficiency we hybridise different modelling techniques into a multiscale platform, with a modular architecture to facilitate model development and validation. The design of this system is provided in the following section.

3.1 Overview of the Platform Model

An overview of the scheme (**Figure 10**) is as follows: *In silico* stromal cells (**Module 1**) are modelled as a series of nodes and edges [21] capable of interacting with lymphocytes and secreting CXCL13 (**Module 2**). CXCL13 diffusion is modelled using a discretized partial differential equation (PDE) [22]. Individual lymphocytes are distinct entities displaying heterogeneity in receptor expression and activation status; as such we have modelled them as agents that adjust their behaviours with respect to vector and ordinary differential equation-based calculations (**Module 3**). This was achieved through adaptation of a published scheme which explicitly accounts for gradient detection and the dynamics of G-protein coupled receptor (GPCR) expression on the cell surface [23,24]. Within this system agents exist within a continuous environment with chemokine existing in discrete grid spaces. An analysis of an agent-based model (ABM) of the number of effector T cells leaving the LN suggests that a 3D model is required to adequately capture the dynamics of T-cell output from the lymph node during infection. 2D models were shown to underestimate lymph node output because the distance between antigen-presenting cells is overestimated in 2D with respect to 3D [25]. As such 3D was deemed most appropriate to model the efficacy of B cell scanning.

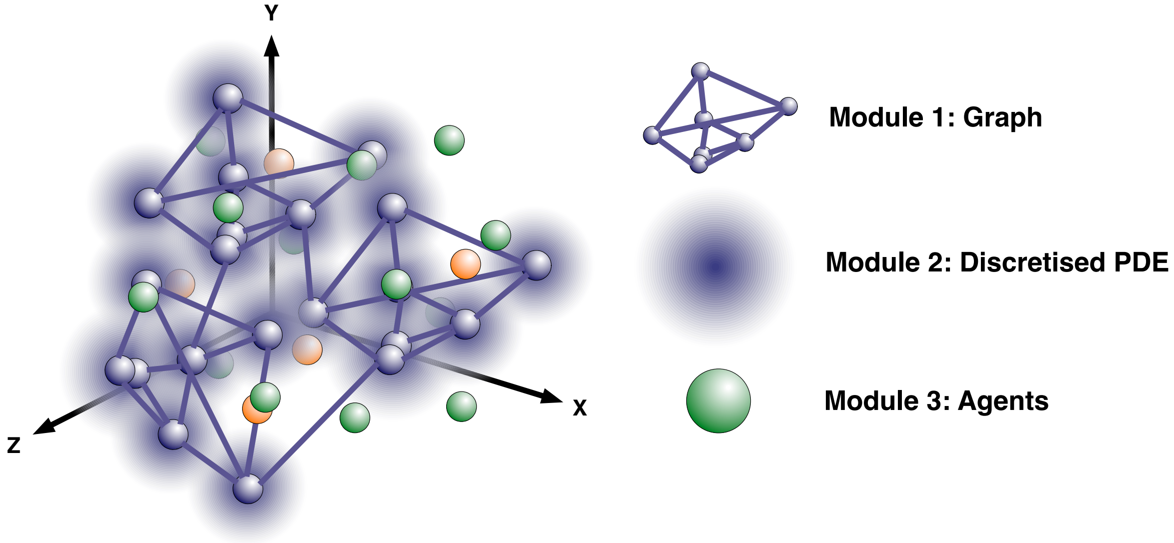

Figure 10. Hybrid 3D multiscale representation of a follicle. In this system stromal cells are modelled as a graph (Module 1), chemokine diffusion is modelled as a discretised partial differential equation (Module 2), while B cells are modelled as rich agents which can interact with their local environment through a set of coupled differential equations and vector based calculations (Module 3).

3.2. Module 1: Stroma

In silico stromal networks are generated using an adaptation of the algorithm developed by Kislitsyn *et al.* (2015). The algorithm stochastically builds a network from an initial node, picking the nearest non-expanded node and generates a set of vectors that will eventually become edges to new nodes. Each vector has a direction and length randomly chosen, but conforming to values derived from experimental data, and the directions are chosen such that the sum of all the vectors in the set is approximately zero. A new node is then created at the end of each vector, and an edge connects them. This process is repeated to generate a graph.

However, this algorithm has some limitations in that it can only describe one stromal subset. To ensure an accurate reconstruction we consider the morphology of different stromal subsets, follicular dendritic cells and reticular cells. In addition, our datasets also show that a number of cell protrusions directly connect to one another, a property which can affect network topology. To account for this, branches between edges are added by creating a vector connecting the midpoints of each edge, subject to subtype-specific constraints on the maximum edge length and the local density such that the degree centrality matches our *in vivo* datasets. This approach is used to generate both RC and FDC networks within the follicle while RCs located just below the subcapsular sinus are stochastically seeded by random sampling of X and Y values subject to density constraints to ensure consistency between *in vivo* and *in silico* edge lengths and degree centralities. To represent the heterogeneity of network structures observed *in vivo* and to ensure no biases were introduced through a specific stromal architecture, the algorithm generates a unique network at the beginning of each simulation run. A suite of automated tests were developed to assess whether edge lengths and degree centralities are within expected bounds and that there are no overlapping nodes or edges.

Using this approach we generate a unique network at the start of each simulation run. Running 250 simulations we find that this approach yields networks with median sigma and omega values of **12.00** and **-0.097** respectively, confirming that the network has small world properties (Figure **11**). The discrepancy between *in vivo* and

in silico sigma values was expected as sigma scales with network size and the *in silico* follicle is approximately 4 times the volume of the tissue section used to perform the topological mapping. Comparison of the median values for both edge lengths and degree centralities for *in silico* and *in vivo* networks shows no statistically significant differences (**Figure 12-13**).

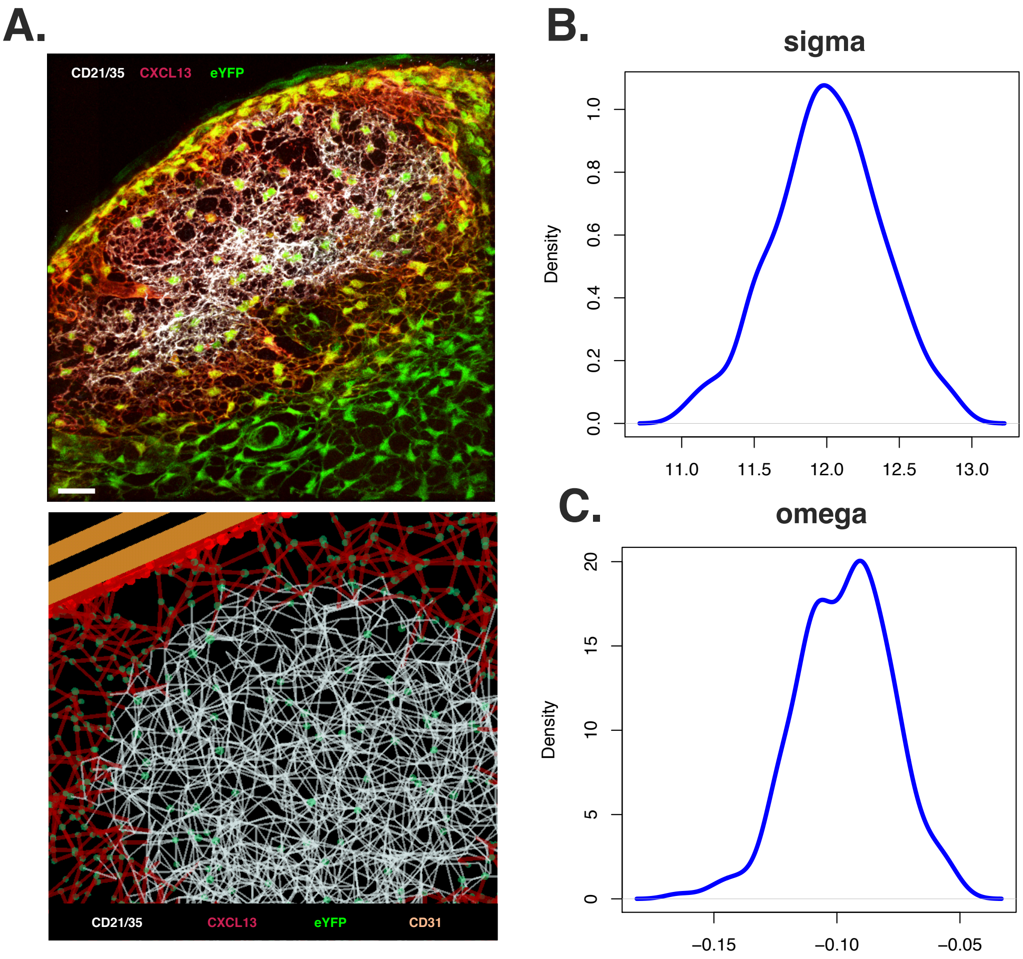

Figure 11. Development and validation of the Stroma module. (a) Top figure shows a lymph node follicle showing tdtomato (red) and eyfp (green) expression from CXCL13-EYFP mice. FDCs are marked with an antibody against CD21/35 (white). Bottom figure shows in silico stromal networks. (b) This figure shows the distribution of sigma values obtained under baseline parameter values from 250 simulation runs. (c) This figure shows the distribution of omega values obtained under baseline parameter values from 250 simulation runs. Scale bar = 50μm. Java code was developed by J.C and S.J.

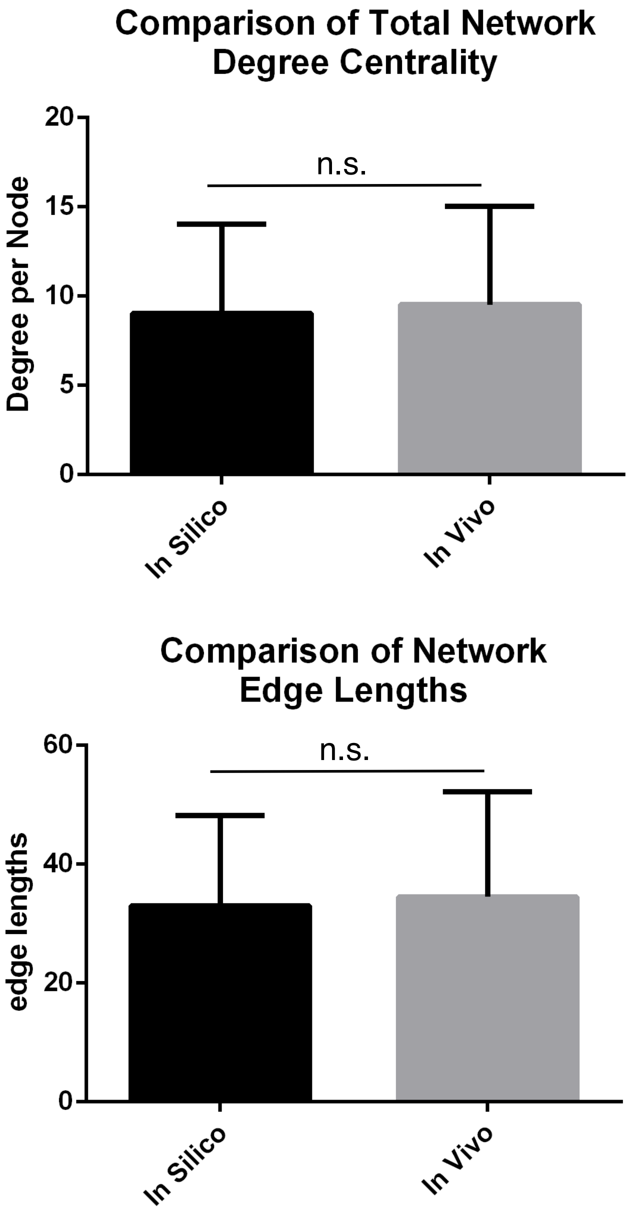

Figure 12. Comparison of *in vivo* and *in silico* edge lengths and degree centralities for the entire follicular network. No statistically significant differences were found between the median values for *in silico* and *in vivo* datasets as determined by a Mann-Whitney test with $p < 0.05$ representing a statistically significant result. Bars represent the median values and error bars represent the I.Q.R.

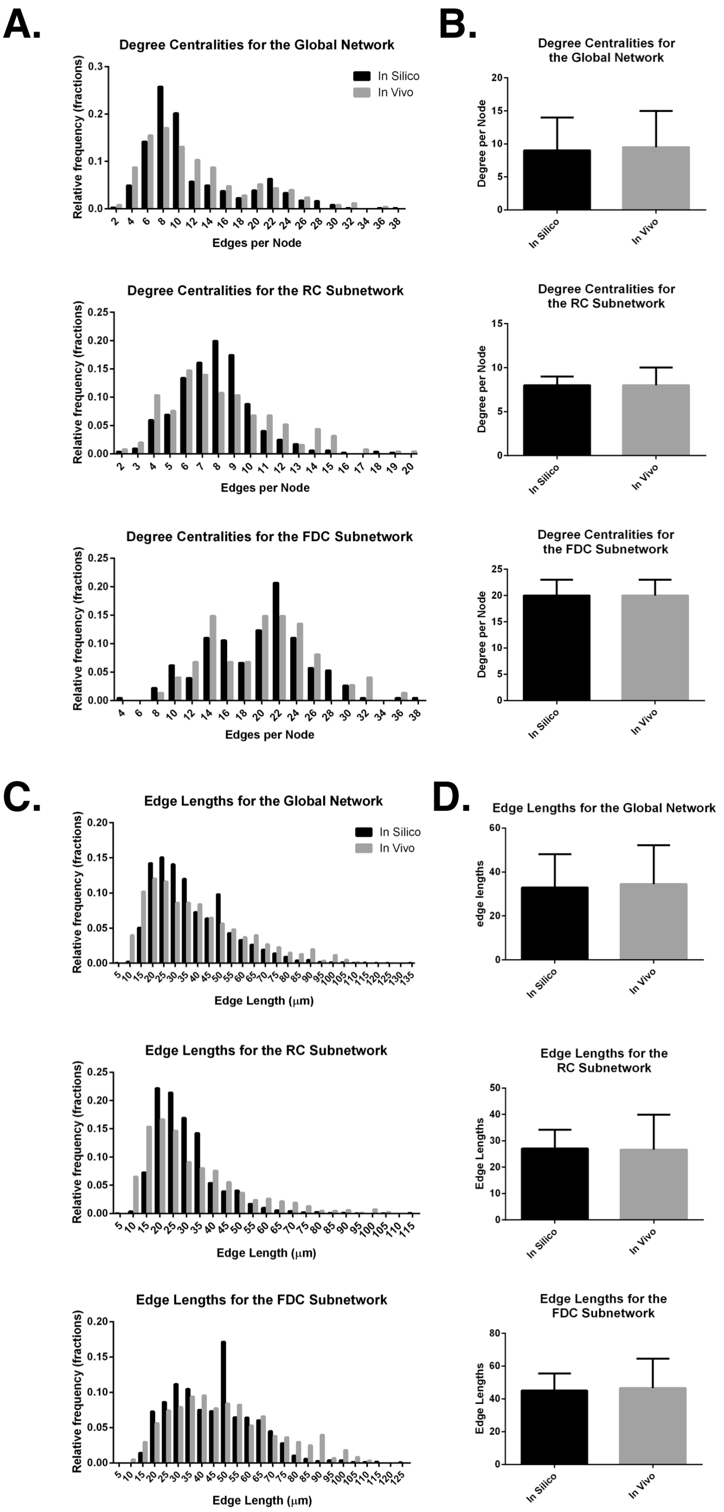

Figure 13. Comparison of *in vivo* and *in silico* follicular networks. (A and C) Comparison of in vivo and in silico distributions of degree centrality and edge length values for the global network and associated subnetworks.(B and D) No statistically significant differences were found between the median values for *in silico* and *in vivo* degree centralities or edge lengths for the global network or associated subnetworks. Significance assessed using a Mann-Whitney test with $p < 0.05$ representing a statistically significant result.

3.3. Module 2: Chemokine

Many different techniques exist to model molecules *in silico,* each with associated advantages and limitations. A common approach to model diffusion is through functions that relate molecular concentration to distance from a source [1], or by PDEs (**Figure 14**) (Bocharov et al., 2011; Guo et al., 2008). As they describe molecular concentrations on a continuous scale at the population level, it can be difficult to incorporate complex behaviours such as localised binding effects into PDEs and distance-concentration functions. At the other extreme, it may also be intractable to model molecules (which exist in much larger numbers and move on faster scales than immune cells) using individual-based approaches. This limitation can make it difficult to simulate the dynamics of chemokine field formation where molecules simultaneously undergo production, diffusion, decay, binding and scavenging, key mechanisms required to shape functional chemotactic gradients [28]. In cases where high model granularity is required at the molecular level, soluble factors can be represented as floating point values on discretised grids (**Figure 14**). The scheme we implement is a discretised form of the heat equation [22]. This mathematical construct is capable of isotropic diffusion[1], can diffuse to an arbitrary number of neighbours and is applicable to linear, planar, spatial and n-dimensional implementations. These attributes make it well suited to studies of molecular components of the immune system (**Figure 14**).

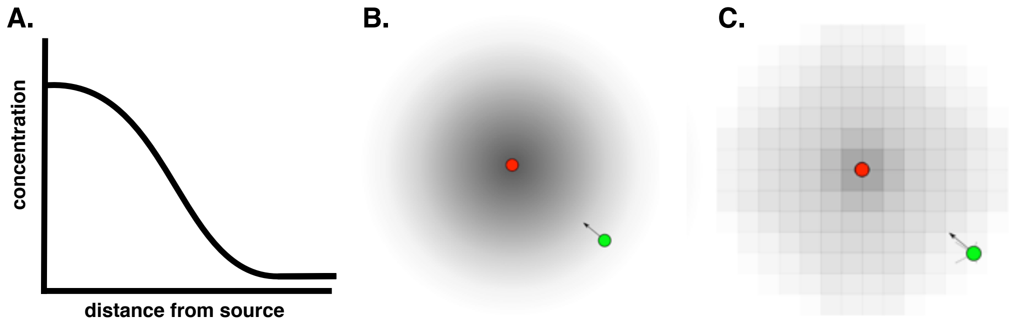

Figure 14. Different schemes to model diffusion in theoretical models. (A) Functions that relate concentration to distance from a source. (B) PDEs predict changes in concentration over time and space on a continuous scale. (C) Discrete PDEs in which the environment is binned into discrete gridspaces; within each gridspace concentration is homogeneous.

---

1 Anisotropy is an implementation artifact where diffusion occurs faster in certain directions than others, making the diffusion neighborhood look square

In this scheme, chemokine molecules diffuses through a discrete 3D environment where the number of moles of chemokine molecules in each grid space *(x,y,z)* is denoted *φ(x,y,z)*. The change in the spatial distribution of molecules is then subject to the following simultaneously occurring processes *(i) production (ii) diffusion (iii) decay and (iv) consumption*. As we are using agents to model individual cells, terms (i) and (iv) emerge from the simulation. Chemokine is secreted by each stromal cell at a fixed rate and is removed from the grid at a rate that is proportional to its current value λ.

$$\varphi(x, y, z)_{t+1} = (1 - \lambda)\varphi(x, y, z)_t$$

where *t* represents the time step. At each discrete time step chemokine diffuses to the grid spaces adjacent and diagonally adjacent to each grid space. The coefficient for the amount of chemokine diffused to each grid space is:

$$A\big(\varphi(x) - \varphi(y)\big)e^{\frac{-d_y^2}{\mu}}$$

$$\mu = 4Dt$$

where chemokine in grid space *x*, $\varphi(x)$, is being diffused to grid space *y*, d_y^2 is the distance squared between *x* and *y*, *D* is the diffusion constant, *t* is the time step, and A is a normalizing constant that ensures the total amount being diffused is less than or equal to the amount that exists:

$$A \sum_{y=1}^{n} e^{\frac{-d_y^2}{\mu}} = 1$$

for an arbitrary grid space where n represents the number of surrounding grid spaces in the diffusion neighbourhood, which in CXCL13Sim is a 3D Moore neighbourhood with 26 neighbouring grid spaces. This approach mitigates an artefact known as anisotropy, where diffusion appears "square" because treating each grid space equally would favour diffusion diagonally due to the larger distance to the corner neighbours

than the lateral ones. This implementation explicitly takes the distance d_y^2 between gridspace x and its adjacent grid space y into account to avoid this effect without the need to implement a tessellated hexagonal grid, which can only be done in 2D. The borders of the grid follow Dirichlet boundary conditions.

When modelling diffusion in discrete space, the speed at which chemokine diffuses is limited by the time step and size of the diffusion neighbourhood. Increasing the size of the diffusion neighbourhood would allow greater diffusion coefficients without a smaller time step, but would significantly increase the time resources required to run the algorithm. To account for this, the time step we use is $\frac{\delta^2}{kD}$ where δ is the length of an edge of the discretized grid spaces, D is the diffusion coefficient, and k is a constant, empirically determined in order to match the measured diffusion speed with the mathematically derived value for mean-squared displacement for a given diffusion constant. The diffusion grid is updated every second while agents are updated once every minute and are assumed quasi-static with respect to diffusion. Automated tests were developed to ensure that chemokine is conserved when the decay constant is set to zero and no agents are in the simulation, that the diffusion coefficient input gives the expected mean-squared displacement output and that the rate of diffusion per time step cannot extend beyond the diffusion neighbourhood.

The baseline value of the diffusion constant was informed using preliminary measures of CXCL13 mobility in follicles of lymph node tissue sections obtained using the single-molecule super-resolution imaging approach described in Miller and Cosgrove (2018). To assess whether the discrepancy between our baseline value of 7.6 microns squared per second and the final published value of 6.6 microns squared per second would effect emergent in silico cellular behaviours we performed a local sensitivity analysis (see figure 30). Results from this analysis show that changing the diffusion constant value between 6-8 microns squared per second did not lead to a statistically significant alteration in cellular behaviours *in silico* and consequently 7.6 was used as the baseline value for the diffusion constant.

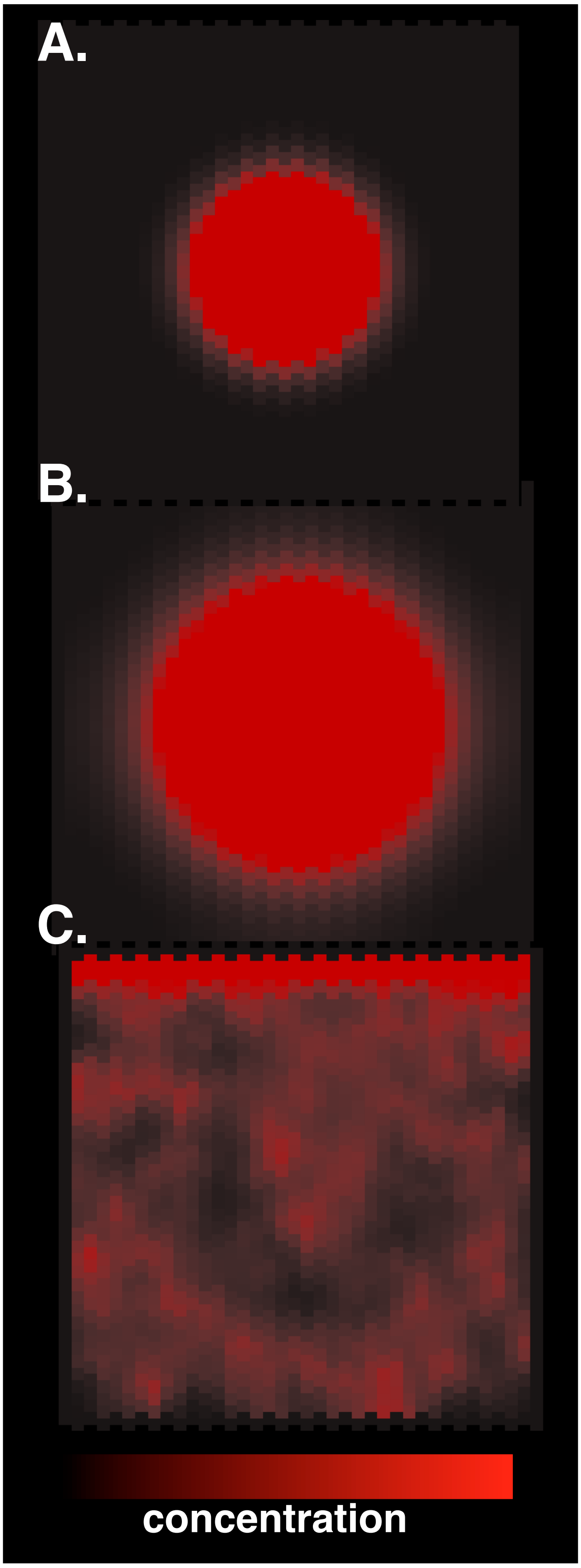

Figure 15. Examples of gradients formed using the Gradjeanu scheme. (A) a chemokine field with low diffusion rate and high decay rate leading to a small circle of CXCL13 expression (red) (B) a chemokine field with high diffusion rate and low decay rate leading to a larger CXCL13 field (C) shows a complex chemokine field generated by a stromal network generated by the algorithm described in section 5.2.2. Java code was developed by S.J and J.C.

3.4. Module 3: Lymphocytes

3.4.1. Lymphocyte Migration

Chemotaxis, and chemokine receptor internalisation and recycling are key mechanisms governing the fine-tuning of responses to chemokines *in vivo* [23,28,29]. In addition, the follicle is a highly dense structure and so it is important to account for interactions between cells. To model these phenomena *in silico,* we adapt the scheme developed by Lin *et al* [23,24]. In this scheme an agent samples local chemokine concentrations using 6 sampling pseudopodia (**Figure 16**). At each pseudopod there are a population of receptors on the cell surface [R_f] that are free to bind ligand [L] at a rate K_{on}. Receptor dynamics are controlled by a set of ordinary differential equations solved on a per agent basis using a 4th order Runge-Kutta scheme [30]. Ligation of the chemokine to its respective receptor [LR] leads to downstream signalling cascades and localisation of actin with ligand dissociating at a rate K_{off}. Following binding receptors are desensitized at a rate K_{des}, internalised at a rate K_i and are recycled at a rate K_r (**Figure 16**). The values for K_{on} and K_{off} are set so that the affinity constant (K_d), that is the ligand concentration at which half of the receptors are bound, is set to 10nM, the baseline concentration within the simulator (**Figure 17**). To quantify the influence of these parameters on the ODE outputs we performed a global sensitivity analysis using latin hypercube sampling and partial rank correlation coefficients PRCCs, a robust measure for quantifying non-linear relationships between parameter inputs and output cell behaviours (Marino et al., 2008). This analysis was performed in ASPASIA, a toolkit for evaluating interventions on systems biology markup language (SBML) model behaviours (**Figure 18**) [31]. It is important to note that by looking at the rank correlation does not take the magnitude of the change of model outputs into account. From calculating [LR] at each pseudopod, a gradient vector, $\overrightarrow{LR}$ is calculated across the cell along 3 axes with ρ representing each individual pseudopod. If $\overrightarrow{LR}$ exceeds a threshold then the cell will become chemotactic, with the overall orientation vector of the cell $\overrightarrow{LR_{total}}$ taken as a sum of $\overrightarrow{LR}$ with a leading edge vector$\overrightarrow{LR_m}$ that accounts for the orientation of the cell from the previous time step.

$$\overrightarrow{LR} = \left([LR_{\rho1}] - [LR_{\rho4}]\right) + \left([LR_{\rho2}] - [LR_{\rho5}]\right) + \left([LR_{\rho3}] - [LR_{\rho6}]\right)$$

$$\overrightarrow{LR_{total}} = \frac{\alpha\overrightarrow{LR} + \overrightarrow{LR_m}}{\left|\alpha\overrightarrow{LR} + \overrightarrow{LR_m}\right|}$$

$$\frac{d[\mathrm{LR}]}{dT} = K_{on}[L][R_f] - K_{des}[LR] - K_{off}[LR]$$

$$\frac{d[R_f]}{dT} = K_r[R_i] - K_{on}[L][R_f] + K_{off}[LR]$$

$$\frac{d[R_i]}{dT} = K_i[R_{des}] - K_r[R_i]$$

$$\frac{d[R_{des}]}{dT} = K_{des}[LR] - K_i[R_{des}]$$

In the presence of chemotactic gradients actin flows polarize at the leading edge of the cell thus the relative weighting between $\overrightarrow{LR}$and $\overrightarrow{LR_m}$ is scaled by a constant α to represent the persistence of the cell; the value of α is dependent on the chemotactic state of the agent. As a universal coupling exists between actin flows and cell speed [32] and relate the increase in velocity $v*$ observed during chemokinesis to cell persistence α using the following expression:

$$v * = \frac{LN(\alpha)}{\gamma}$$

$$\alpha = \begin{cases} \alpha_1, & if\ chemotactic \\ \alpha_2, & if\ not\ chemotactic \end{cases}$$

The value of the scalar term γ was determined empirically, by fitting the model to WT migration data and verifying against CXCR5$^{-/-}$ migration patterns [9]. A number of automated tests were developed to ensure that total receptor values are conserved over time, and that agents move towards high concentrations of chemokines when expressing CXCR5.

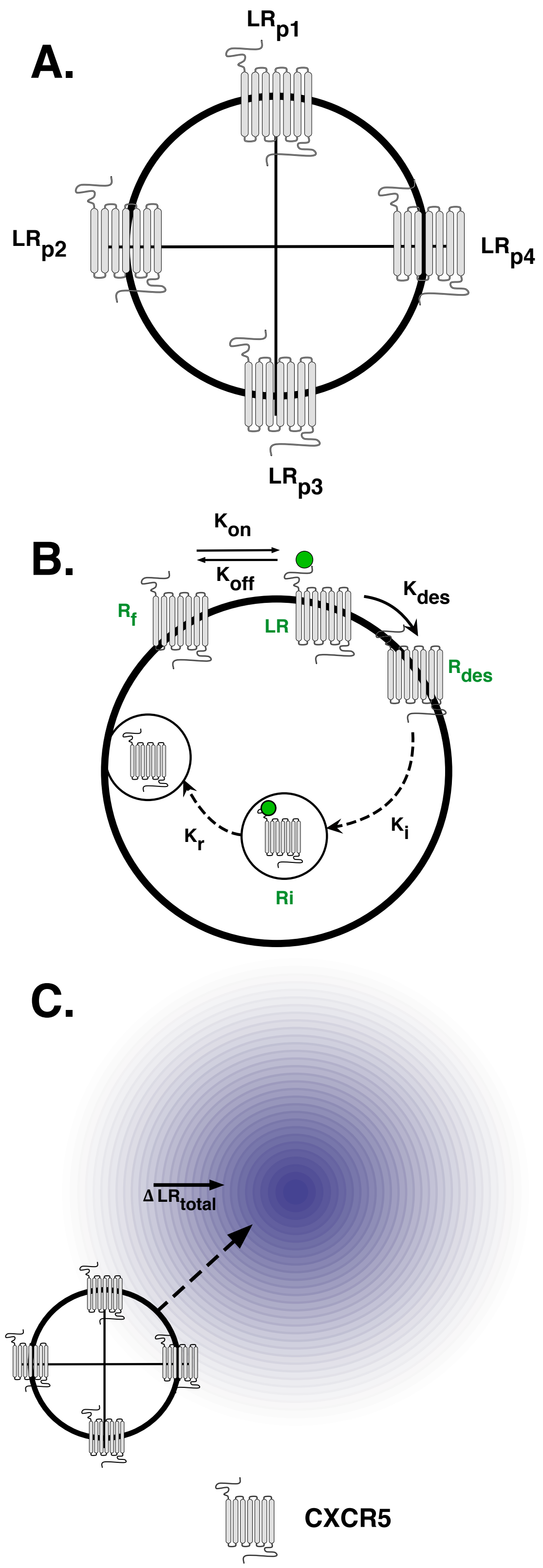

Figure 16. Overview of receptor and migration kinetics in CXCL13Sim. Each cell has 6 chemokine sampling pseudopods (4 are shown). At each pseudopod, signalling is a function of local chemokine concentrations and receptor expression. Receptor expression is dynamic and subject to ligand association/dissociation as well as receptor desensitization, internalization and recycling. From the amount of receptors signalling at each pseudopod, gradient vectors are calculated along 3 axes. The overall net movement vector LRtotal determined by summing these vectors with a polarity vector representing cell persistence in a given direction.

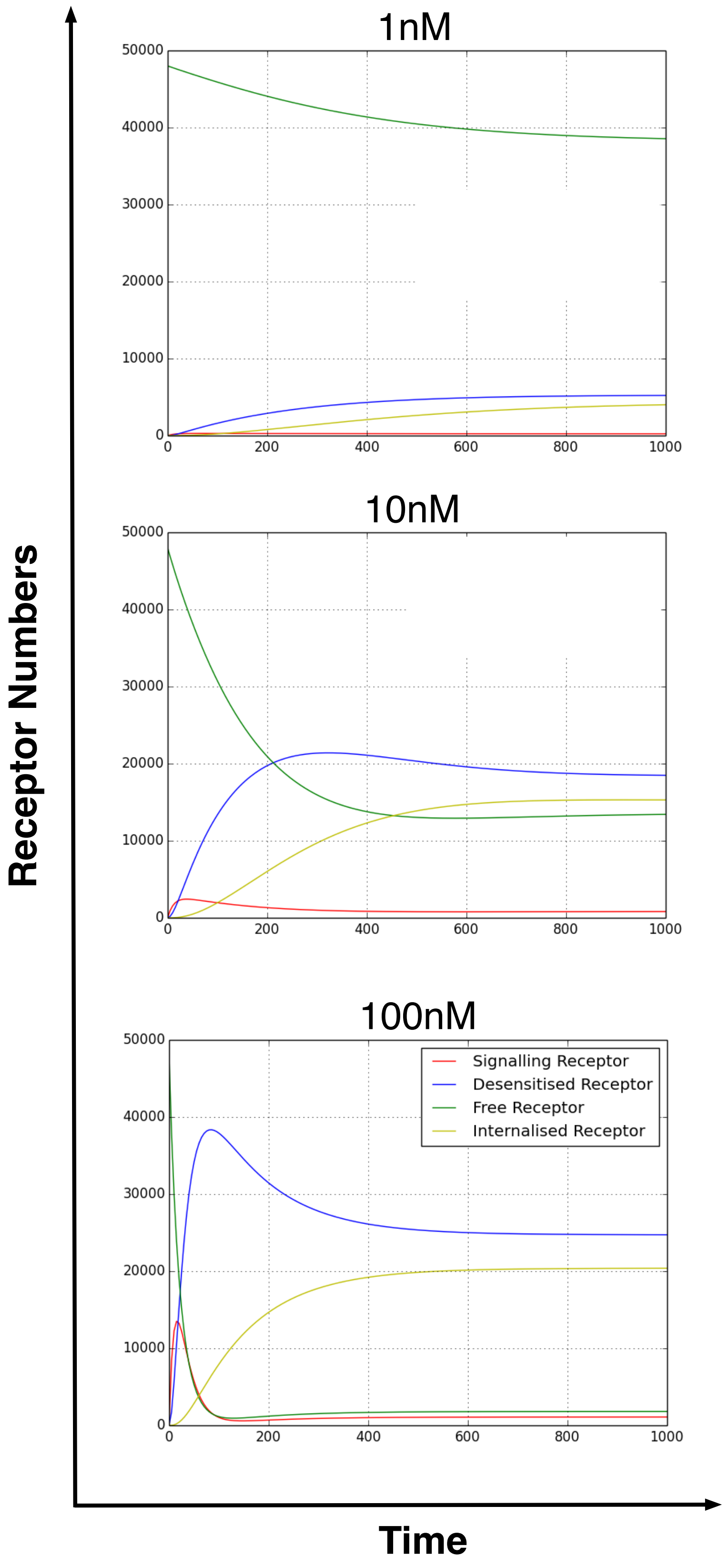

Figure 17 CXCR5 Kinetics for 1nM, 10nM and 100nM CXCL13. Receptor kinetics with calibrated parameter values in response to 1nM show large numbers of free receptors on the cell surface with relatively low levels of desensitised, internalised and signalling receptors. As the amount of chemokine increases, the amount of free receptors decreases and the number of desensitised receptors increases.

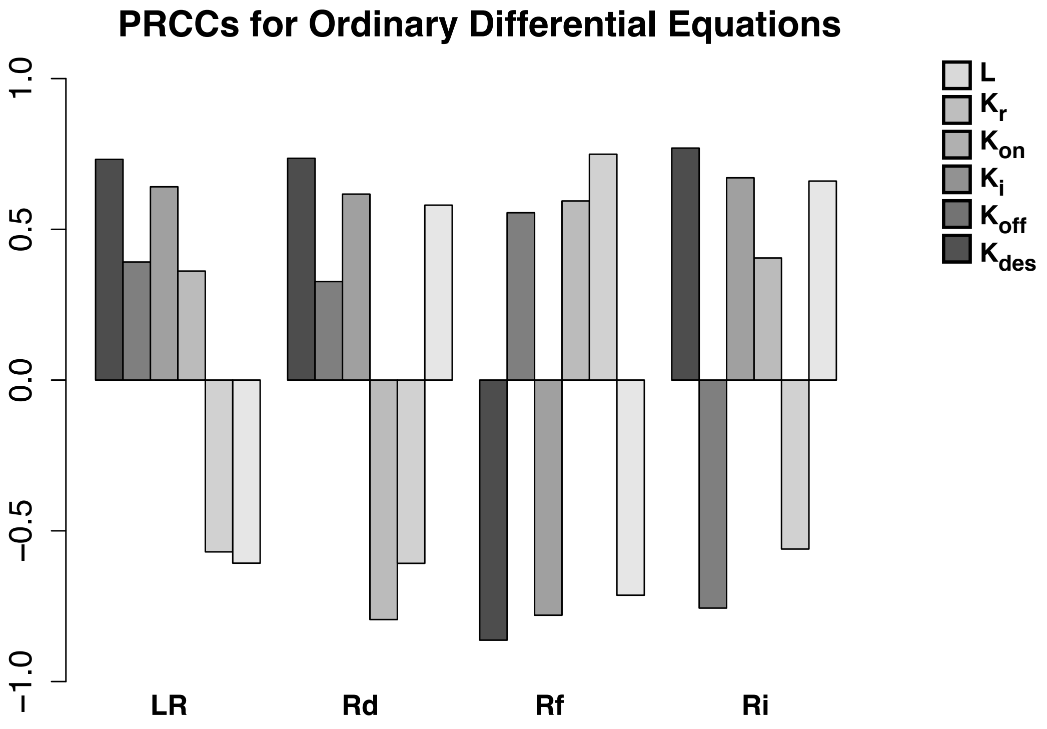

Figure 18. Parameter sensitivities for ODE parameters performed using ASPASIA [31]. The influence of a parameter on model outputs is quantified using a partial rank correlation coefficient.

3.4.2 Lymphocyte Interactions

To account for dense lymph node environment lymphocyte migration must take into account interactions with other cell types. As time proceeds in fixed discrete intervals we treat both the movement vector of lymphocytes and the edge of the stromal cell as lines (**Figure 19**). To determine if the two agents are interacting we calculate whether the closest point between the two lines is less than the sum of their diameters. To determine the closest point, we define the lines L1 and L2 as follows:

$$\mathrm{L1(s)} = \mathrm{P1} + \mathrm{sd1}, \text{where } \mathrm{d1} = \mathrm{Q1} - \mathrm{P1} \quad (5.13)$$
$$\mathrm{L2(t)} = \mathrm{P2} + \mathrm{td2}, \text{where } \mathrm{d2} = \mathrm{Q2} - \mathrm{P2} \quad (5.14)$$

Then for some pair of values for s and t, L1 (s) and L2 (t) correspond to the closest points on the lines, and v(s,t) describes a vector between them.

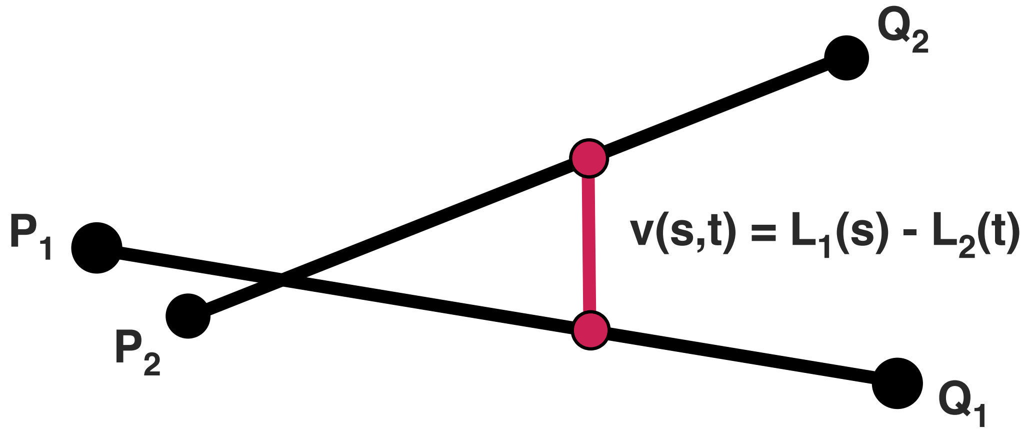

Figure 19. Modelling lymphocyte interactions with stroma. In this scheme the movement vector of lymphocytes and stromal cell processes are modelled as lines. Each line is defined by two points, Pi and Qi. To determine if the two agents are interacting we calculate whether the closest point between the two lines is less than the sum of their diameters. Then for some pair of values for s and t, L1 (s) and L2 (t) correspond to the closest points on the lines, and v(s,t) describes a vector between them.

The closest point between the two lines is obtained when the vector is perpendicular to both lines (**Figure 19**) i.e. when the dot product of the two vectors is equal to zero or

$$d1 * v(s,t) = 0$$
$$d2 * v(s,t) = 0$$

Using Cramer's rule we can then solve this system of equations to determine which values s and t where L1(s) and L2(t) correspond to the closest points on the lines. Additionally, cells may interact with each other, however cell structure is dynamic and cells are observed to slide over one another. To account for this, once an agent has determined the direction in which to move, the probability that the cell can move towards the target location is determined as $e^{-\delta}$, where δ is the number of cells in the target location. Automated tests were developed to ensure that lymphocytes completely caged within a tight network of stromal cell protrusions cannot pass through due to interacting with the network, and that the number of agents per gridspace does not exceed a threshold value.

3.5 Integration of Model Subunits

System architecture is modelled using an adaptation of the UML as per the domain model. This specification defines how model subunits interface and details the flow of information through the system (**Figure 20 – 24)**. On the diagrams, the modules are specified using: *M1, M2* and *M3* to represent stroma, chemokine and lymphocytes respectively. Key decisions and abstractions made during the development process are presented as arguments over evidence[2] using an adaptation of goal structuring notation.

---

2 Available from https://www.york.ac.uk/computational-immunology/software/cxcl13sim/

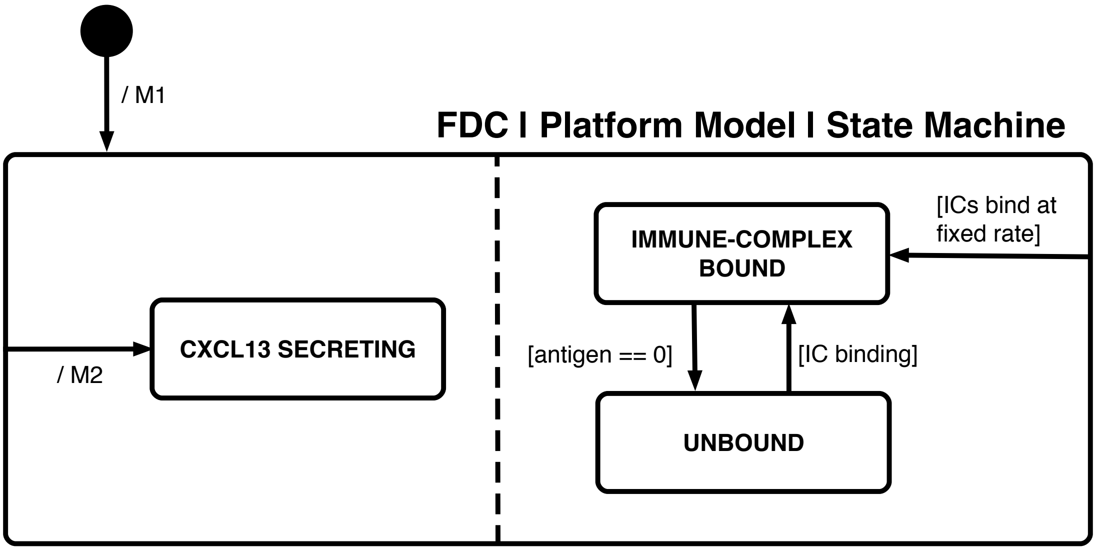

Figure 20. State machine diagram for a Follicular Dendritic Cell. FDCs are resident in the system at t0 and are generated using the algorithm described in M1. FDCs secrete chemokine at a fixed rate and once secreted, chemokine diffuses as described in M2. Antigen levels are expressed as integers and are decreased following interactions with cognate B cells. LTβR mediated stimulation of CXCL13 production is assumed constant in homeostatic conditions and is thus not explicitly referenced in the Platform Model.

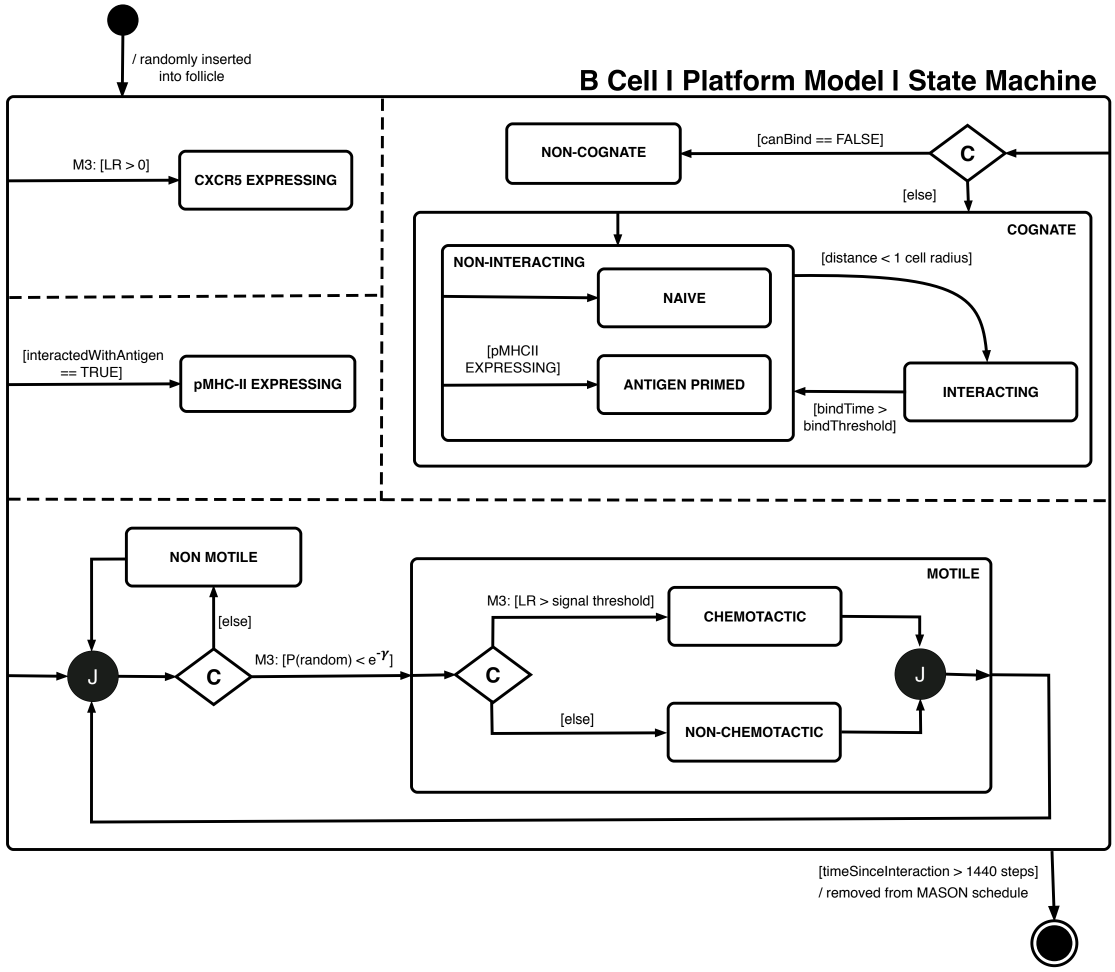

Figure 21. State machine diagram for a B cell. B cells are seeded randomly in the follicle at the start of the simulation in a cognate or non-cognate state on the basis of antigen specificity. For a cognate cell to become primed it must be within 1 cell radius of cognate antigen. With respect to M3, a B cell may change into a CXCL13 desensitised state if the magnitude of the cells orientation vector does not exceed a threshold value. To migrate, a B cell must determine if there is space to move and will then move either randomly or in the direction of a gradient on the basis of it sensitivity to ligand.

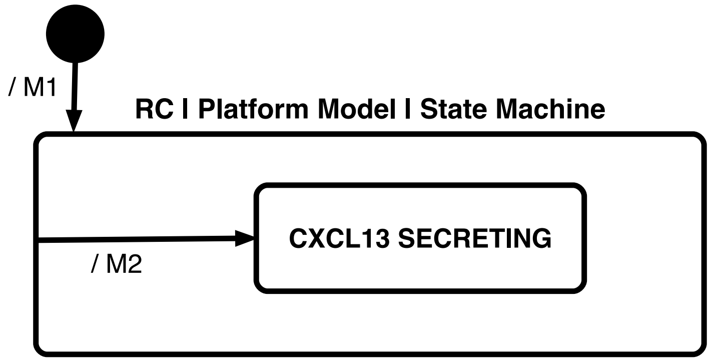

Figure 22. **State machine diagram for RCs.** RCs are resident in the system at the start of the simulation and are generated using the algorithm described in M1. They secrete chemokine at a fixed rate and once secreted, chemokine diffuses as described in M2.

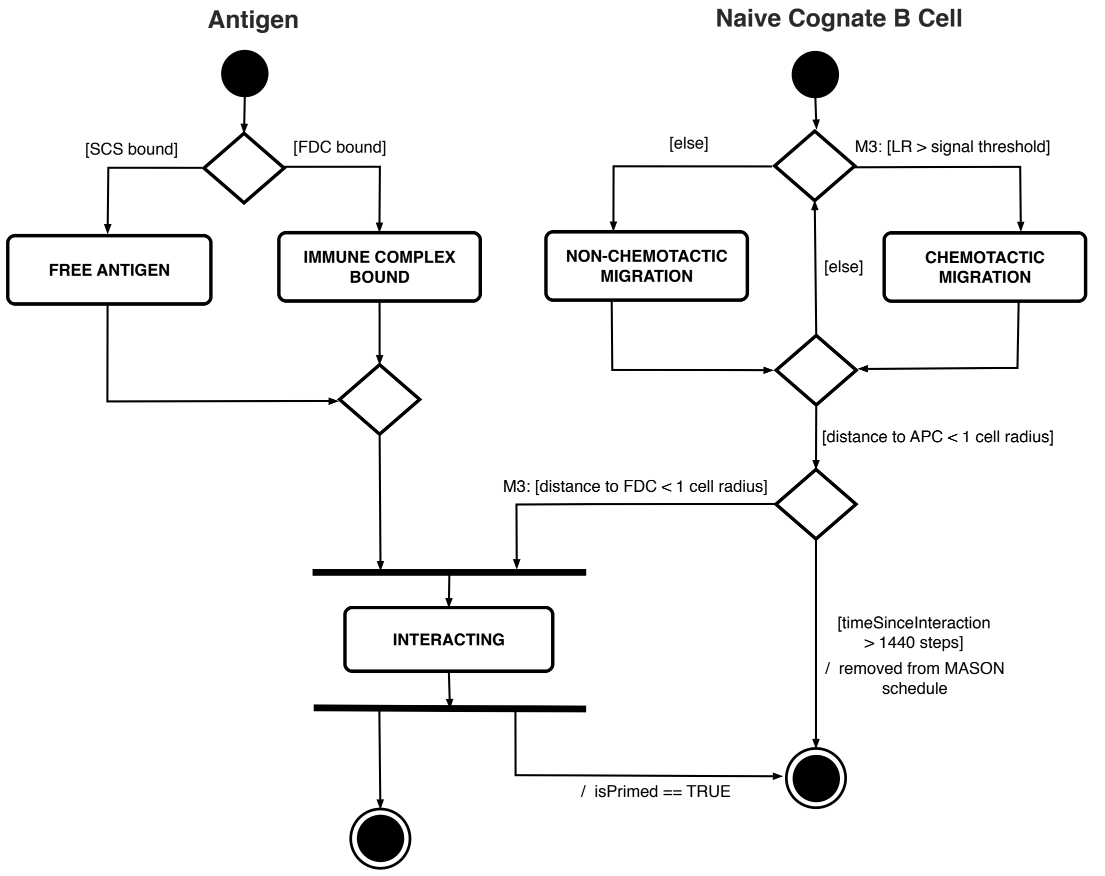

Figure 23. Activity diagram for B cells: At the beginning of the activity FDCs express a fixed number of antigen, specified by an integer value. CXCL13 is secreted by FDCs, diffuses and is removed from the grid as specified in M2. If the number of signalling CXCR5 molecules ([LR]) exceeds a threshold value then the B cell is capable of detecting a chemokine gradient. When the magnitude of the orientation vector LRtotal exceeds a signalling threshold then the cell will orientate towards the gradient otherwise it will orientate randomly. Before migrating a B cell must also determine if it has space available and to become primed the B cell must interact with antigen.

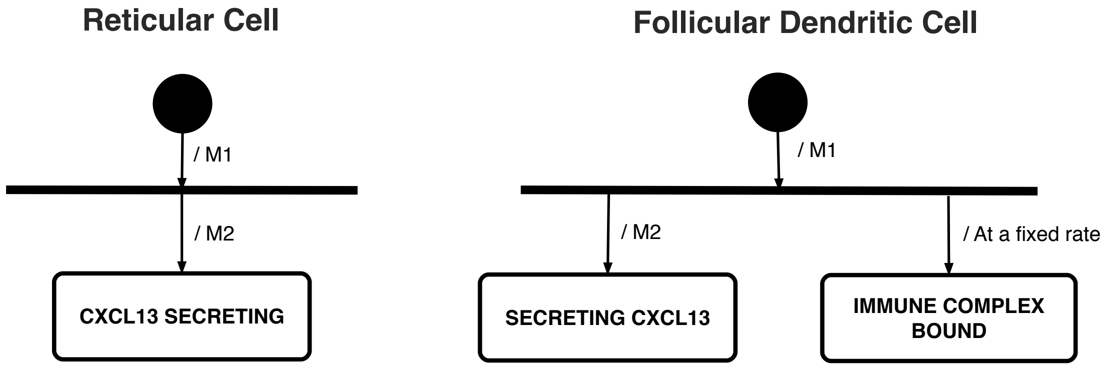

Figure 24. **Activity diagram for stromal cells:** At the beginning of the activity FDCs express a fixed number of antigen, specified by an integer value. CXCL13 is secreted by FDCs, RCs at a fixed rate.

3.6 Model Outputs

Once a simulation run is complete, a summary .csv file is produced with the metrics detailed in **Table 1** for each cognate B-cell agent. These metrics facilitate comparison with experimental measures of migration and are used to assess the influence of parameter perturbations on the emergent cellular behaviours.

Measure	Description
**Total Displacement**	Record the steps taken by cells and calculate displacement over a fixed time period using vector addition.
**Net Displacement**	Euclidean distance between the first and last position of the cell
**Cell Velocity**	Total displacement / time
**Motility Coefficient**	Net displacement^2 / 6* time
**Meandering Index**	√Time * (net displacement / total displacement)
**checkPointsReached**	Number of unique gridspaces reached

Table 1. Summary of model outputs. Following each individual simulation run, the following metrics are calculated for each B-cell agent.

Section 4. Simulation Platform

The simulation platform was implemented using Java and the MASON ABM library version 19 [33] in an iterative process of implementation, validation and refactoring using Acceptance Test-Driven Development (ATDD) [34]. Tests are continually assessed and refined as the project progresses and are incorporated into an automated regression framework using the java library JUnit (available from http://junit.org/junit4/) to ensure that new code does not disrupt existing functionality, expediting the development process. To quantify sources of uncertainty in the our simulator we used the R software package SPARTAN [35]. This package contains a suite of statistical techniques (described in more detail in the following sections) specifically designed to help understand the relationship between the simulator and the physical system it describes.

4.1 Model Calibration and Validation

Within the simulation platform each module was developed using Java and the multi agent simulation library MASON [33]. A total of 125 tests are incorporated into an automated regression-testing pipeline using JUnit (available from http://junit.org/junit4/), and the eclEmma package (available from http://www.eclemma.org/download.html).

To calibrate the simulator, the 13 free parameters were systematically changed and outputs were compared to *in vivo* multiphoton datasets. Fluorescently labelled wild-type and CXCR5$^{-/-}$ B cells were adoptively transferred into WT hosts and their distribution and migration patterns inside popliteal lymph nodes analysed using selective plane illumination microscopy, which preserves the three-dimensional organ structure. The median values for 250 simulation runs are 1.15 for the meandering index, 13.28 for motility coefficient and 8.11 for speed (**Figure 25**). Comparison of a single *In silico* run with *in vivo* datasets showed no significant differences between motility coefficients, meandering indices and velocities for (a) wild-type or (b) CXCR5$^{-/-}$ B cells; assessed using a Mann-Whitney test at a significance level of 5% (**Figure 26**). The additional 9 parameters were fixed at empirically determined or calibrated values representing key attributes such as cell size and the density of the stromal cell network that are not designed to change between simulation runs.

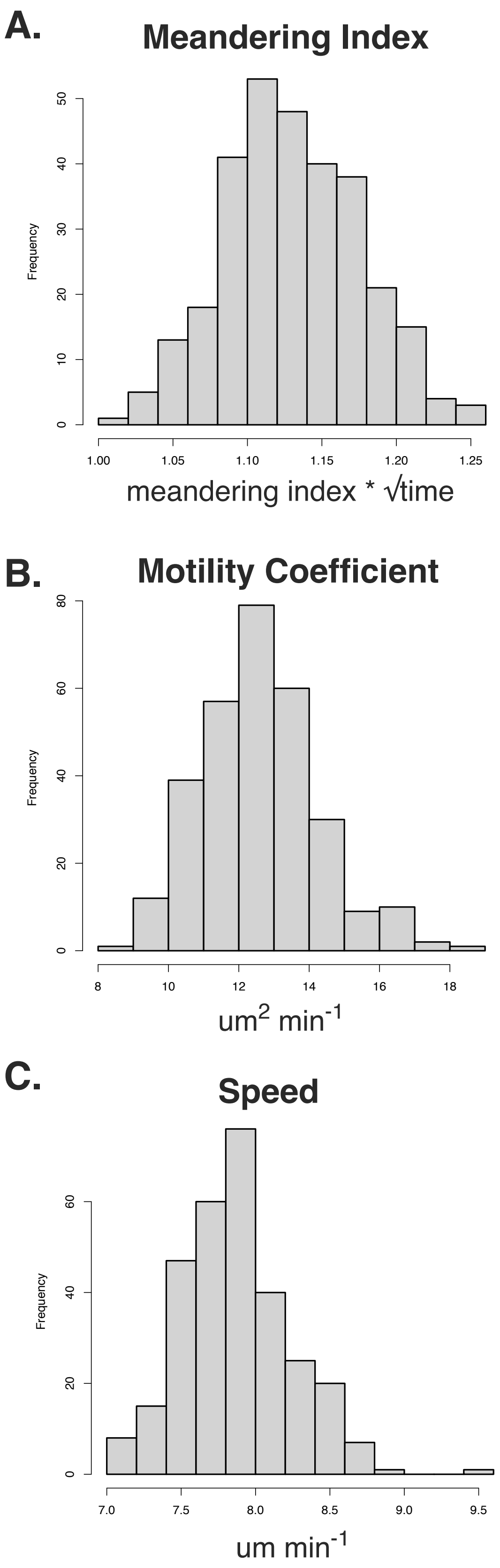

Figure 25. Distributions of emergent cell behaviours under baseline conditions. (A) Distribution of meandering index values for baseline parameter values. (B) Distribution of motility coefficient values for baseline parameter values. (C) Distribution of speed values for baseline parameter values.

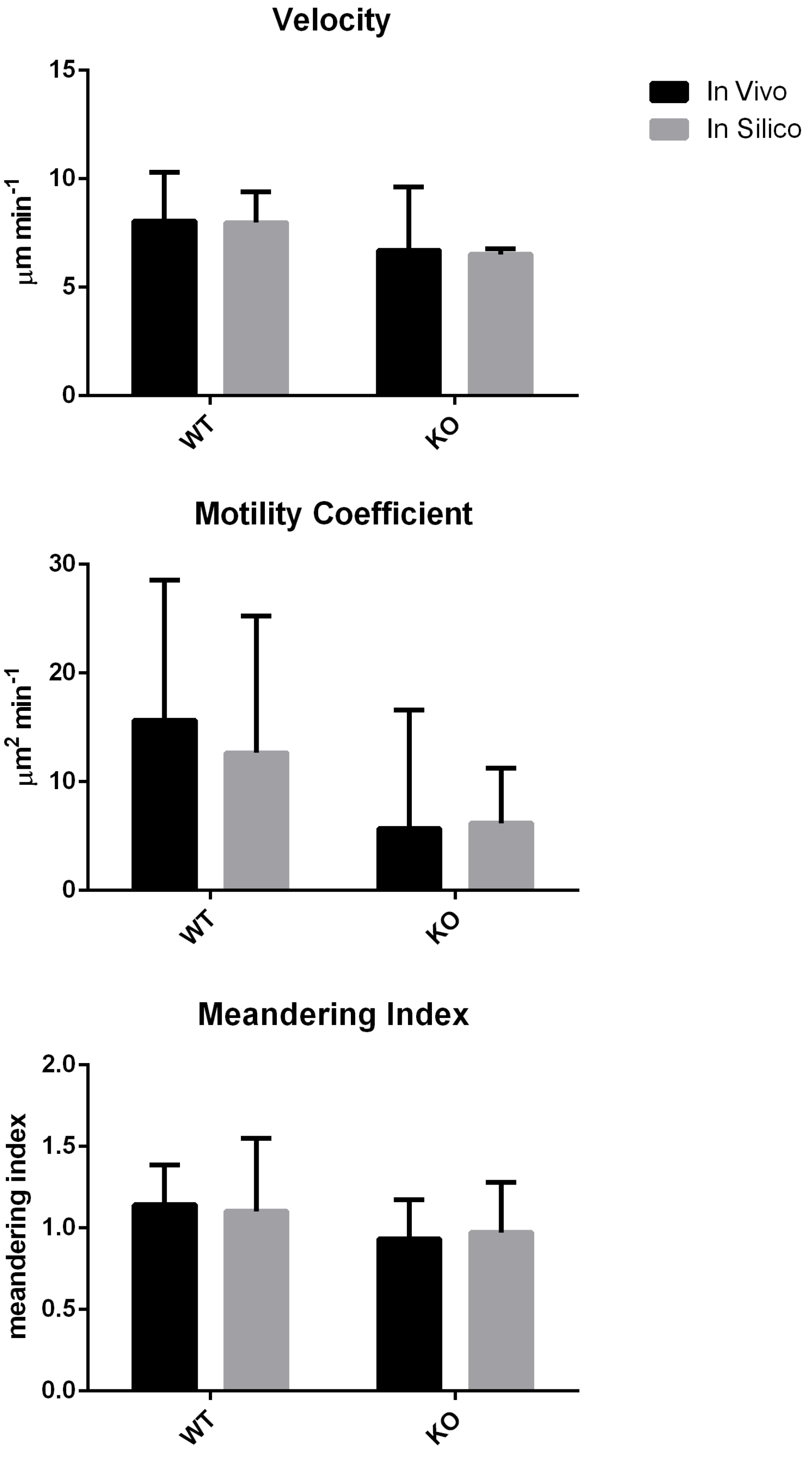

Figure 26. Calibration of emergent behaviours against experimental behaviours. No significant differences were observed between *in silico* and *in vivo* motility coefficients, meandering indices and velocities for wild type or CXCR5$^{-/-}$ B cells. All datasets were non-normal as determined by a Shapiro-Wilk test, subsequently; a Mann-Whitney test was used to determine if datasets were significantly different at a significance level of 5%. Bar charts represent the median value with error bars representing the I.Q.R.

Parameter	Value	Unit	Range	Reference
**B Cell Size**	7	µm	Constant	[36]
**Total Number of B cells**	6000	cells	Constant	Measured
**Total Number of MRCs**	100	cells	Constant	Measured
**Total Number of FDCs**	~200	cells	Constant	Measured
**Total Number of BRCs**	~450	cells	Constant	Measured
**Proportion of Cognate Cells**	5	%	Constant	-
**Displacement constant**	7.4	µm min^{-1}	[1-10]	Calibrated
**Signal threshold**	10	ΔLR	Constant	[37,38]
**Maximum turn angle**	180	Degrees	Constant	[9]
**Total receptor number**	48,000	Receptors	[10,000-100,000]	[39]
$\mathbf{K_{on}}$	4.8 x 10^5	M s^{-1}	[$1x10^5$-$1x10^6$]	[40]
$\mathbf{K_i}$	0.0033	s^{-1}	[0.001-0.01]	[39,41]
$\mathbf{K_{des}}$	0.075	s^{-1}	[0.01-0.1]	[39,41]
$\mathbf{K_r}$	0.004	s^{-1}	[0.001-0.01]	[39,41]
$\mathbf{K_{off}}$	0.0048	s^{-1}	[0.001-0.01]	[39,41]
**FDC secretion rate**	0.18	fg min^{-1} $cell^{-1}$	[0.1-0.5]	[42,43]
**RC secretion rate**	0.18	fg min^{-1} $cell^{-1}$	[0.1-0.5]	[42,43]
**CXCL13 decay rate**	0.007	s^{-1}	[0.0002-0.05]	[44,45]
**CXCL13 diffusion rate**	7.6	$µm^2$ s^{-1}	[0-146]	Measured
**Polarity**	0.475	-	0-1	Calibrated
**Random Polarity**	3.8	-	Constant	Calibrated

Table2. Summary of parameter values. For each parameter the name, baseline value and range used for uncertainty and sensitivity analyses is provided. Parameter values were determined experimentally or in cases where no direct experimental value exists, upper and lower limits were derived from indirect evidence, baseline values were then determined by fitting the model to experimental datasets (calibration). The model was further validated against migration data from $CXCR5^{-/-}$ B cells and parameters were removed where possible. The values for stromal cells are averaged over 250 runs with individual values varying to a small extent between runs due to stochastic network formation.

5.4 Understanding Aleatory and Parameter Uncertainty

5.4.1 Mitigation of Aleatory Uncertainty

CXCL13Sim is non-deterministic and therefore, repeat experiments using the same parameter set can lead to differing results (**Figure 25**). This variation is termed aleatory uncertainty and because of this effect multiple simulation executions must be performed to obtain a representative result. To determine how many runs are required to give a representative output for a given parameter set we perform an aleatory analysis (detailed further above) [35,46] (**Figure 27**).

In this approach, 20 distributions were generated and contrasted for each sample size. A distribution of median responses for each simulation run is generated for each of the 20 subsets. Distributions 2–20 are contrasted with the distribution from the 1st set using the Vargha-Delaney A-Test (Vargha and Delaney, 2000), a non-parametric effect magnitude test that establishes scientific significance by contrasting two populations of samples and returning the probability that a randomly selected sample from one population will be larger than a randomly selected sample from the other. Values of 0.5 indicate that the medians are the same while values of 1 and 0 mean that there is no overlap. In our analyses we set thresholds for small (0.56), medium (0.66) and large (0.71) effect sizes based on values suggested by [46,47]. We define a statistically bheioavural alteration as one that exceeds the large effect size threshold.This analysis shows that large differences between parameter sets can be detected with 50 replicate run for all outputs while smaller differences require 250 runs.

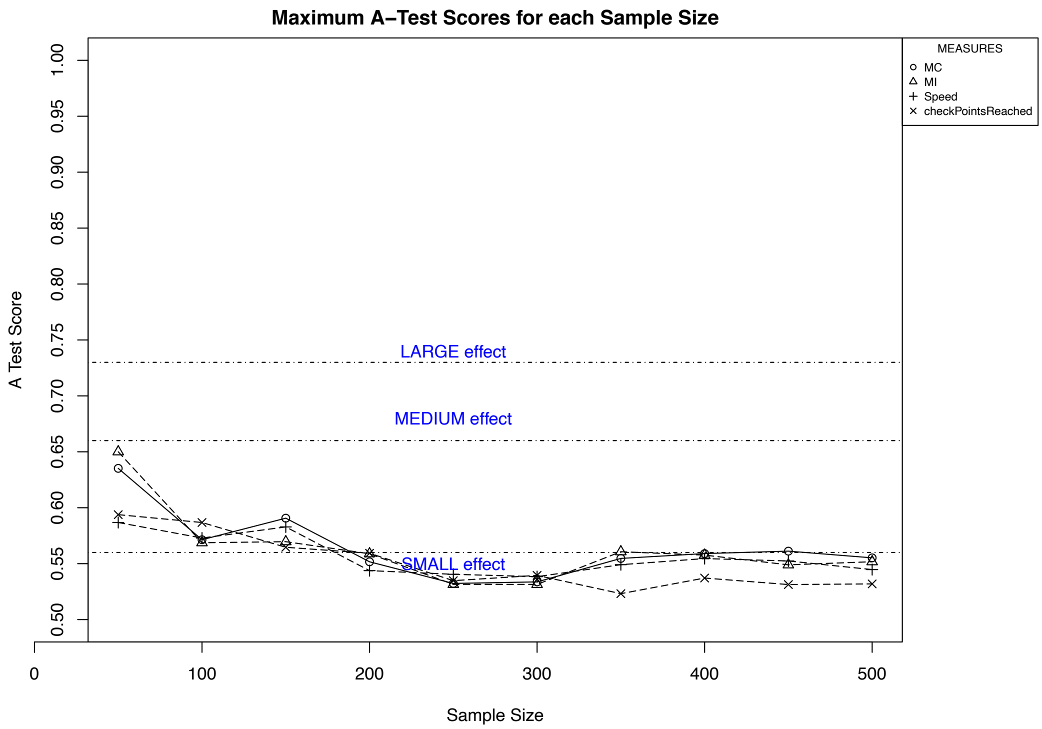

Figure 27. **Mitigating aleatory uncertainty.** An aleatory uncertainty analysis was performed using the SPARTAN package in R. This analysis shows that 250 runs are required to provide enough power to detect small effects in all model outputs. Based on this analysis parameter sensitivity analyses were run with 50 replicates while *in silico* experiments are run with 250 replicates. checkPointsReached (the amount of unique grid spaces reached within a simulation run) ✖; meandering index (MI) Δ; motility coefficient (MC) **o**; speed **+**.

5.4.2 Local Sensitivity Analysis

To quantify parameter uncertainty in CXCL13Sim we first perform a local parameter SA using the SPARTAN statistical package as follows: each parameter is adjusted within the ranges specified in **Table 2**, with all other parameters remaining at their calibrated value with 100 replicates used for each parameter set to mitigate aleatory uncertainty. The Vargha-Delaney A-Test described above is employed to determine if changing the parameter value has led to significant difference in comparison with baseline behaviours (Vargha and Delaney, 2000).

In this analysis we divided parameters into 3 groups. Parameters controlling B cell migration (B-cell displacement constant and cell polarity) had a significant impact on both cell migration and scanning rates (**Figure 28**). Analysis of parameters controlling CXCR5 expression (K_{des}, K_{off}, K_r, K_i, K_{on} and R_f) (**Figure 29**) show a high level of uncertainty around parameters K_{off}, K_r, K_i with perturbations not leading to a large difference in cell migration and scanning rates. However, K_{des}, K_{on} and R_f were influential in regulating cell migration. Only perturbed rates of K_{des} altered the rate of scanning (**Figure 29**). Perturbations to parameters controlling CXCL13 expression (cell secretion rates, diffusion constant and decay rate) led to significant changes in both cell behaviours and scanning rates (**Figure 30**).

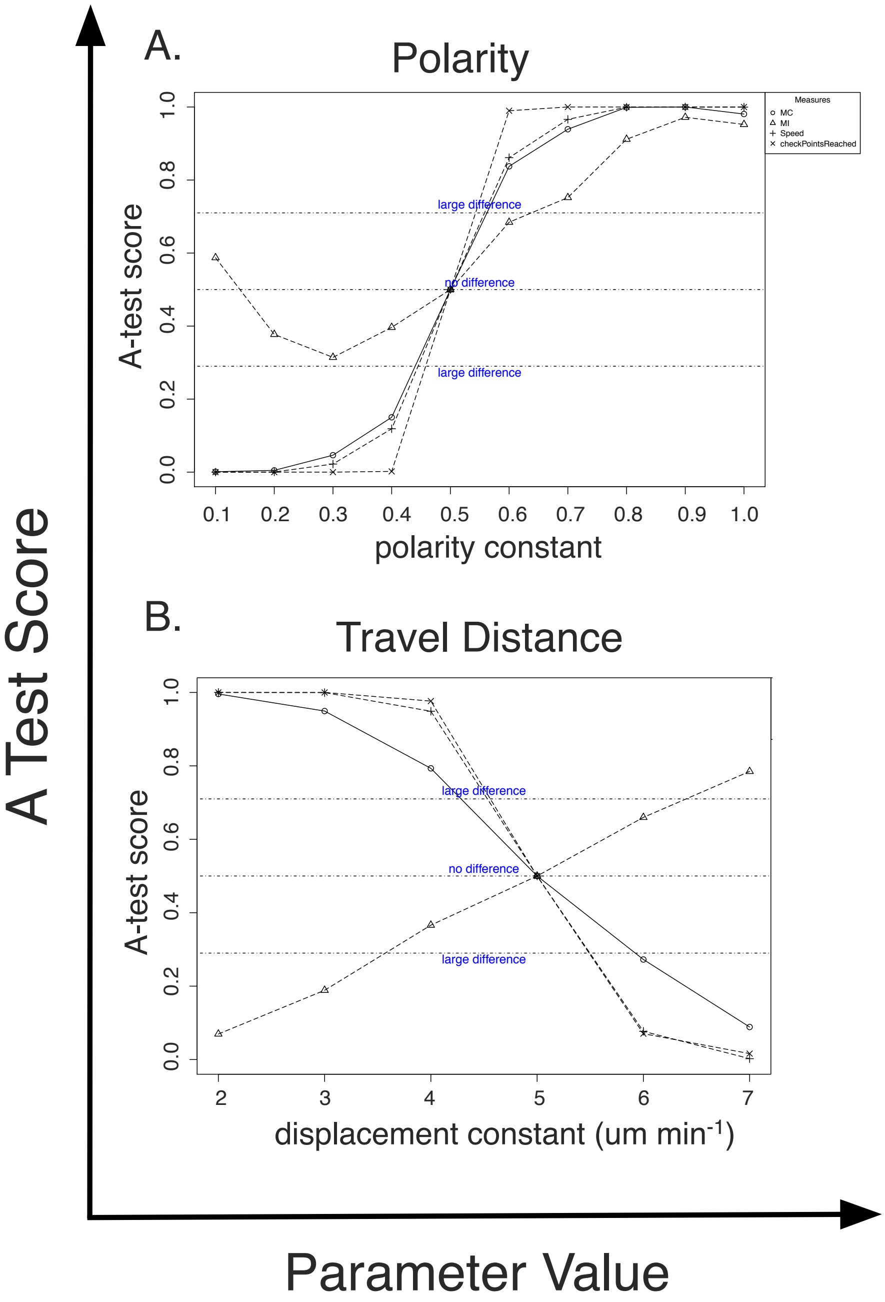

Figure 28. A-test scores when OAT adjusting parameters which relate to B-cell migration and numbers. Parameters *travel distance and polarity* were incrementally changed within their likelihood distributions using an OAT parameter robustness approach. A significant alteration in simulation outputs from baseline behaviours was determined using the Vargha-Delaney A-Test. checkPointsReached ×; meandering index (MI) Δ; motility coefficient (MC) **o**; speed **+**.

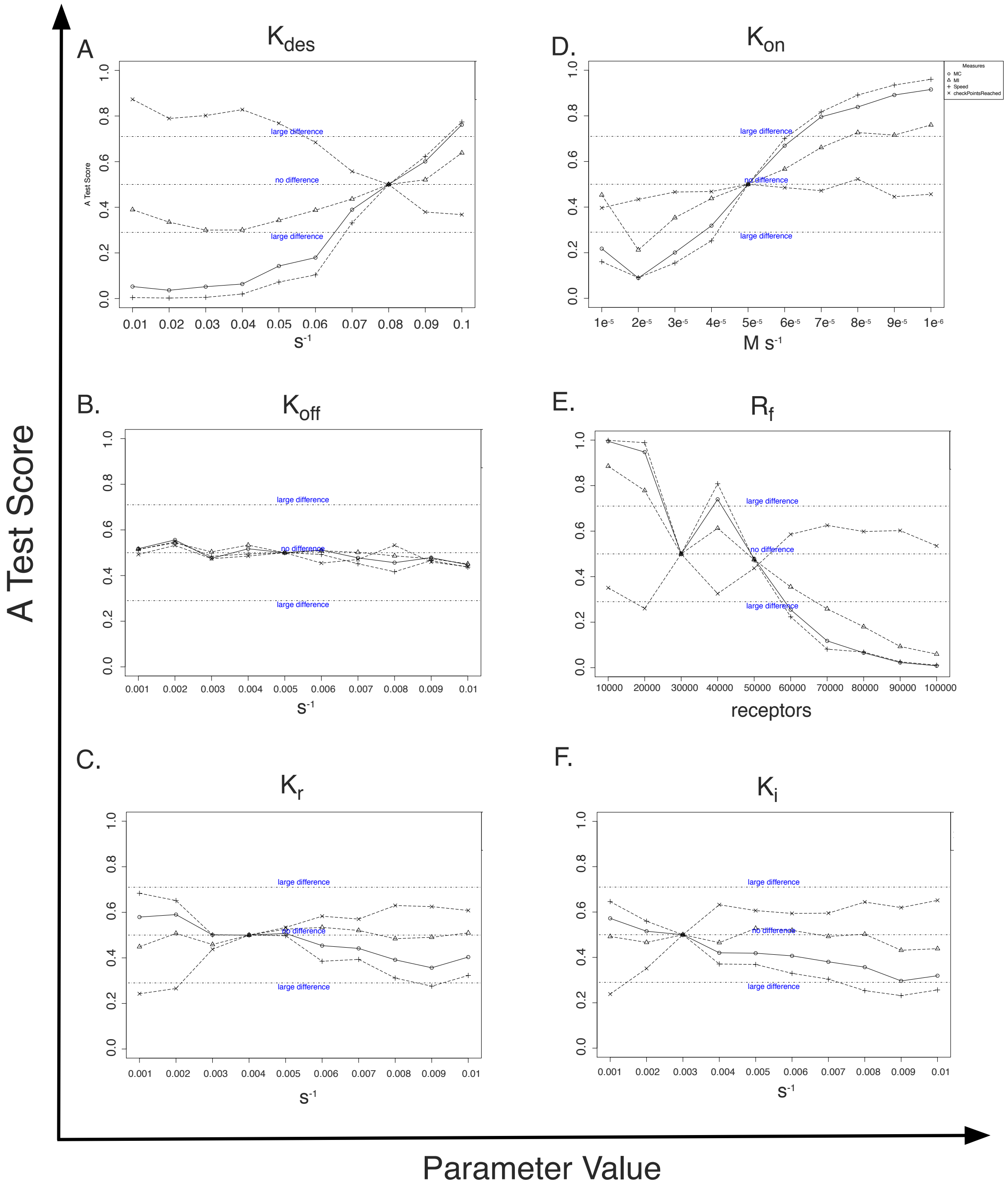

Figure 29. A-test scores when OAT adjusting parameters which relate to CXCR5 expression. Parameters K_{des}, K_i, K_a, K_{off}, K_r and Rf were incrementally changed within their likelihood distributions using an OAT parameter robustness approach. A significant alteration in simulation outputs from baseline behaviours was determined using the Vargha-Delaney A-Test. checkPointsReached ×; meandering index (MI) Δ; motility coefficient (MC) **o**; speed **+**.

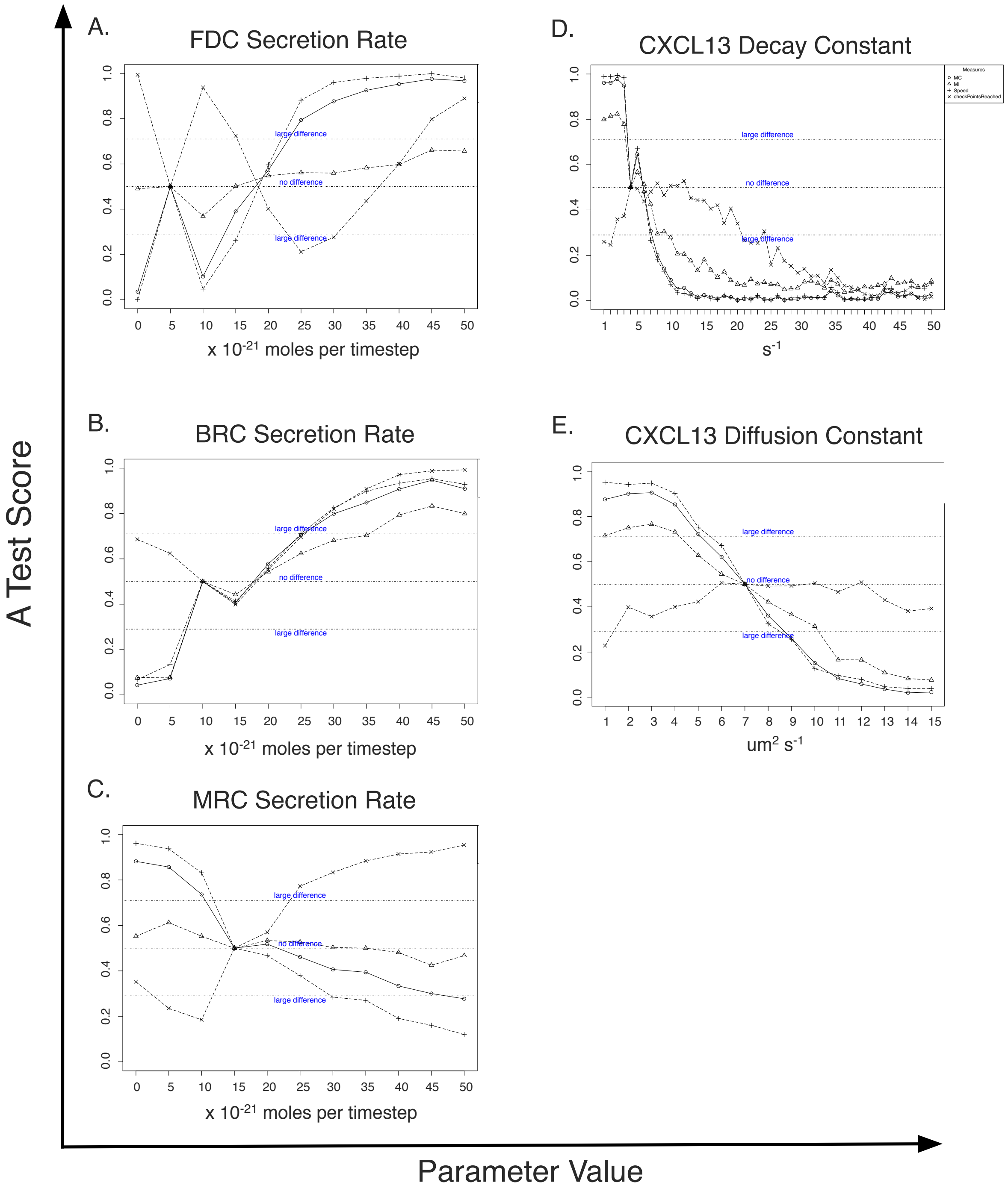

Figure 30. A-test scores when OAT adjusting parameters which relate to CXCL13 bioavailability. Parameters *diffusion coefficient, cxcl13 emitted* and *decay constant* were incrementally changed within their likelihood distributions using an OAT parameter robustness approach. A significant alteration in simulation outputs from baseline behaviours was determined using the Vargha-Delaney A-Test. checkPointsReached ×; meandering index (MI) Δ; motility coefficient (MC) **o**; speed **+**.

5.4.3 Global Sensitivity Analysis

To assess combinatorial effects of parameter perturbations we performed a global sensitivity analysis using latin hypercube sampling that partitions the distribution of each input parameter into intervals of equal probability, selecting one sample from each interval (**Figure 31-32**). LHC sampling generated 1000 parameter sets, 100 executions per parameter set were performed on a high-performance cluster and the influence of each parameter was quantified using a PRCC (detailed in **Chapter 2.2.9**). The parameters polarity, travel distance, and total number of CXCR5 receptors were key determinants of cell migration and scanning rates (**Figure 5.22-5.23**).

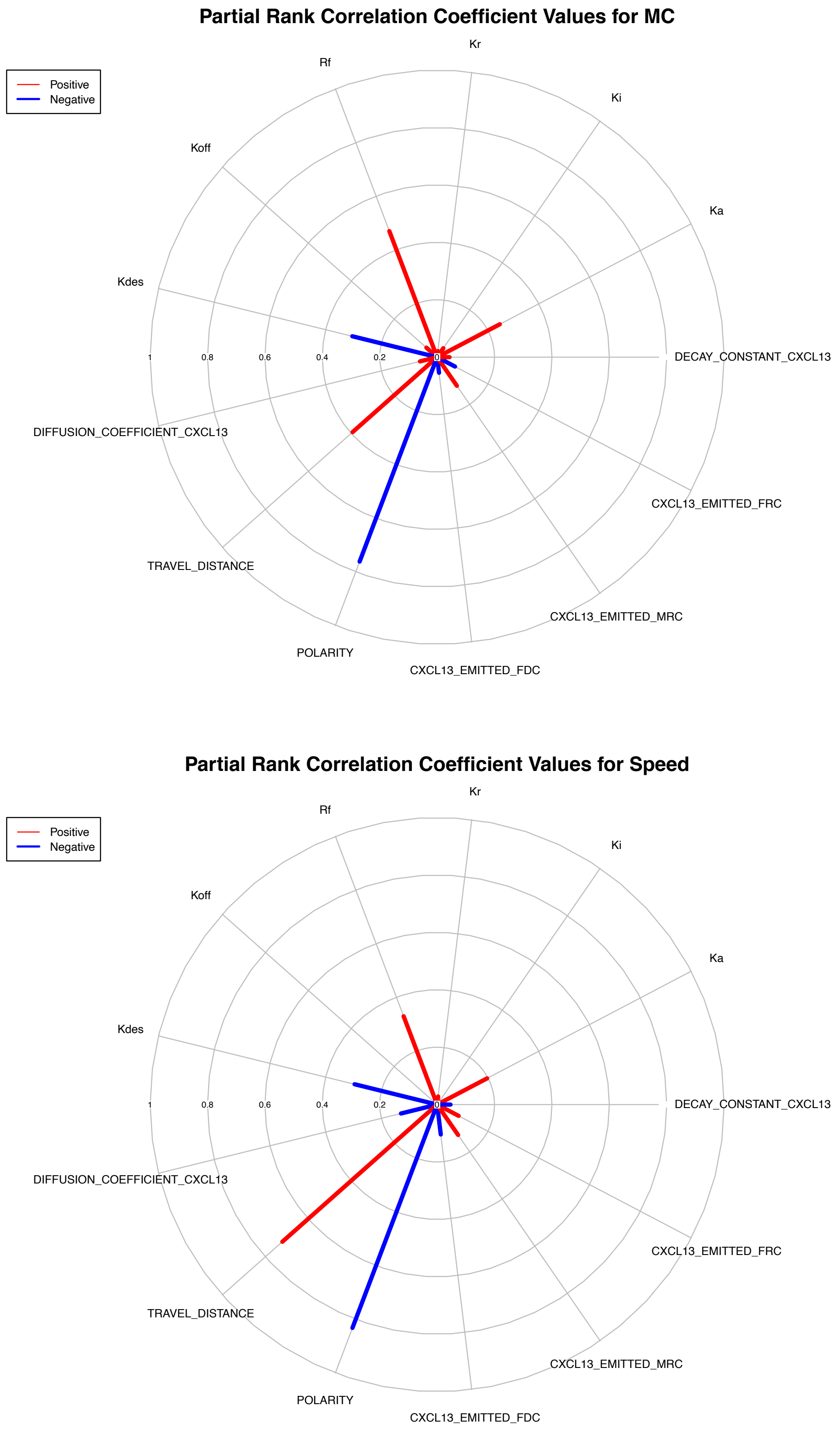

Figure 31. Partial rank correlation coefficients for Motility Coefficient and Speed. Red lines show positive correlations, blue lines show negative correlations. The length of each line represents the magnitude of the correlation between a parameter and an output discounting the linear effects of other parameters

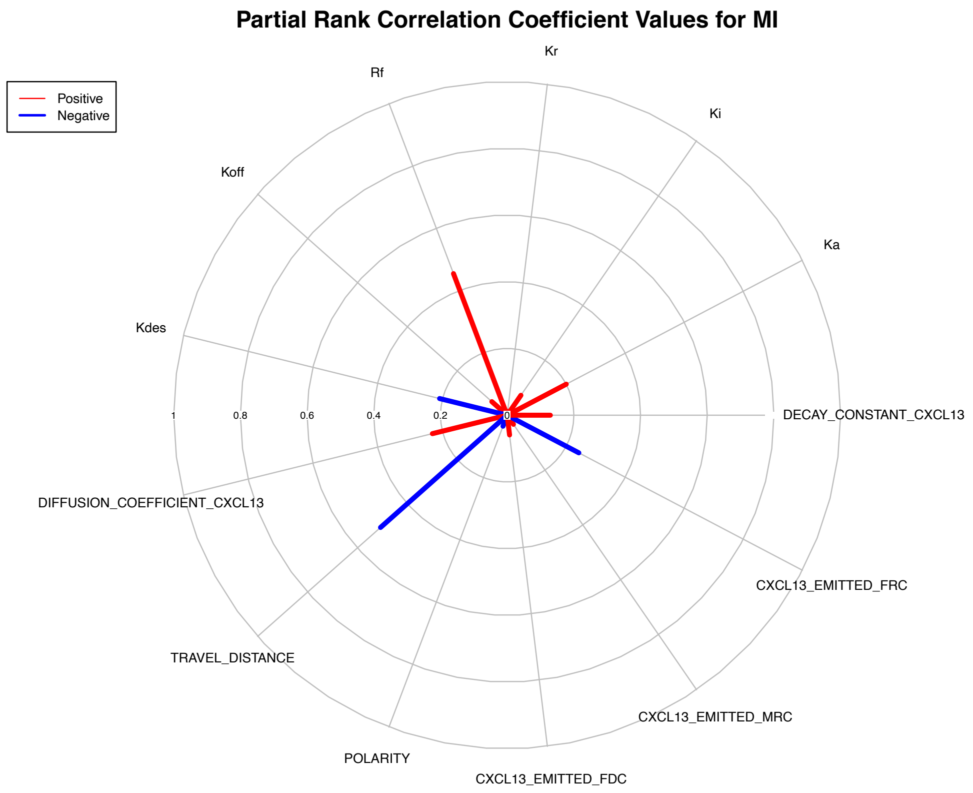

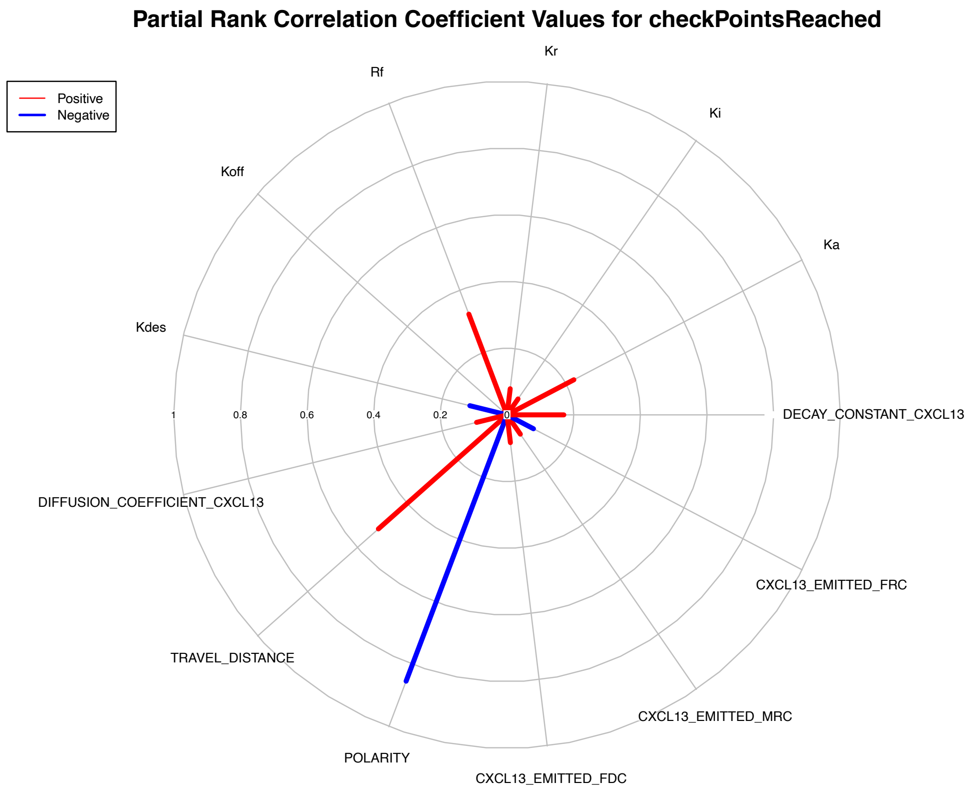

Figure 32. Partial rank correlation coefficients for Meandering Index and checkPointsReached. Red lines show positive correlations, blue lines show negative correlations. The length of each line represents the magnitude of the correlation between a parameter and an output discounting the linear effects of other parameters

Appendix 1. Arguing that the Simulation is a Fit for Purpose Representation of the Biological System

The design and implementation decisions made when constructing a simulator are influenced by the overarching scientific objectives of the work, with simulation results interpreted in this context [4,48]. To argue that the simulator fulfils its remit, acceptance tests, key design decisions, and information used to inform the design, development and validation of the model and simulation are presented as arguments over evidence using a visual notation derived from goal structuring notation (**Figure 33**) and can be opened using the ARTOO tool[3] [4]. This diagrammatic tool facilitates transparency of model design and analysis, capturing the reasoning behind the inclusion or exclusion of each biological feature and recording assumptions, as well as pointing to evidence supporting model-derived conclusions.

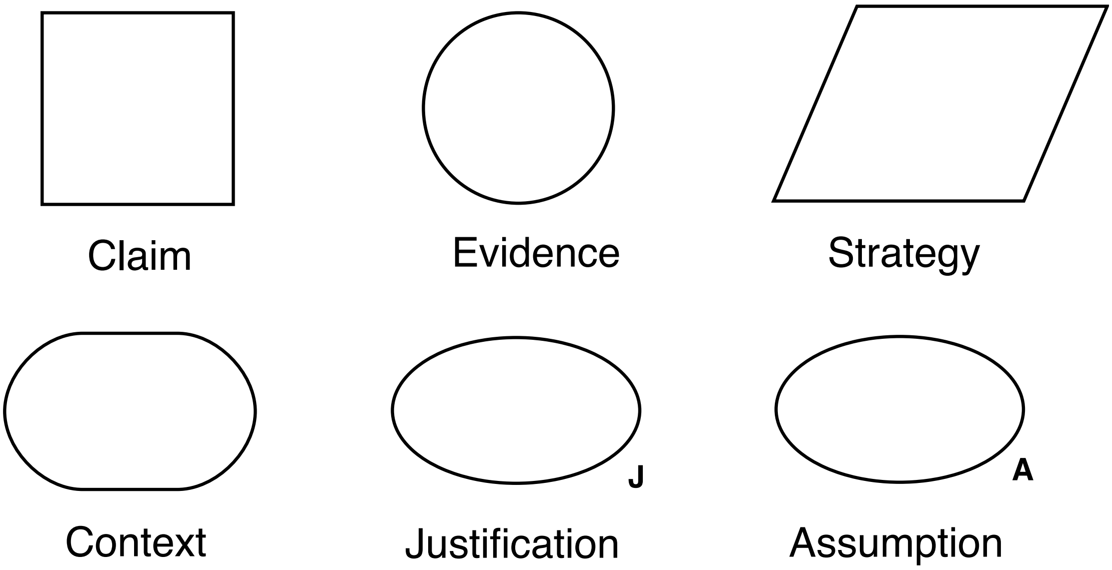

Figure 33. Key for Argumentation Notation: (i) claim the purpose of the argument we are seeking to support; (ii) Evidence that supports the argument made in the attached claim; (iii) strategy the steps that will be taken to argue that the claim is supported; (iv) context defines the purpose of the argument and key terms; (v) justification provides a reasoning behind the selection of a strategy or claim and (vi) assumption provides an explicit statement of any assumptions made in place of biological understanding. Figure reproduced from [4].

---

3 Available from: http://artoofree.simomics.com/

References

1. Alden, K., Timmis, J., Andrews, P. S., Veiga-Fernandes, H. & Coles, M. C. Pairing experimentation and computational modeling to understand the role of tissue inducer cells in the development of lymphoid organs. *Inflammation* **3**, 172 (2012).
2. Bown, J. *et al.* Engineering simulations for cancer systems biology. *Curr. Drug Targets* **13**, 1560–1574 (2012).
3. Read, M., Andrews, P. S., Timmis, J. & Kumar, V. Modelling biological behaviours with the unified modelling language: an immunological case study and critique. *J. R. Soc. Interface* **11**, 20140704 (2014).
4. Alden, K. *et al.* Using argument notation to engineer biological simulations with increased confidence. *J. R. Soc. Interface* **12**, 20141059 (2015).
5. Roozendaal, R., Mebius, R. E. & Kraal, G. The conduit system of the lymph node. *Int. Immunol.* **20**, 1483–1487 (2008).
6. Rantakari, P. *et al.* The endothelial protein PLVAP in lymphatics controls the entry of lymphocytes and antigens into lymph nodes. *Nat. Immunol.* **16**, 386–396 (2015).
7. Roozendaal, R. *et al.* Conduits mediate transport of low-molecular-weight antigen to lymph node follicles. *Immunity* **30**, 264–276 (2009).
8. El Shikh, M. E. M., El Sayed, R. M., Sukumar, S., Szakal, A. K. & Tew, J. G. Activation of B cells by antigens on follicular dendritic cells. *Trends Immunol.* **31**, 205–211 (2010).
9. Coelho, F. M. *et al.* Naive B-cell trafficking is shaped by local chemokine availability and LFA-1–independent stromal interactions. *Blood* **121**, 4101–4109 (2013).
10. Batista, F. D. & Harwood, N. E. The who, how and where of antigen presentation to B cells. *Nat. Rev. Immunol.* **9**, 15–27 (2009).
11. Carrasco, Y. R. & Batista, F. D. B Cells Acquire Particulate Antigen in a Macrophage-Rich Area at the Boundary between the Follicle and the Subcapsular Sinus of the Lymph Node. *Immunity* **27**, 160–171 (2007).
12. Pereira, J. P., Kelly, L. M. & Cyster, J. G. Finding the right niche: B-cell migration in the early phases of T-dependent antibody responses. *Int. Immunol.* **22**, 413–419 (2010).

13. Phan, T. G., Grigorova, I., Okada, T. & Cyster, J. G. Subcapsular encounter and complement-dependent transport of immune complexes by lymph node B cells. *Nat. Immunol.* **8**, 992–1000 (2007).

14. Grigorova, I. L., Panteleev, M. & Cyster, J. G. Lymph node cortical sinus organization and relationship to lymphocyte egress dynamics and antigen exposure. *Proc. Natl. Acad. Sci.* **107**, 20447–20452 (2010).

15. Matloubian, M. *et al.* Lymphocyte egress from thymus and peripheral lymphoid organs is dependent on S1P receptor 1. *Nature* **427**, 355–360 (2004).

16. Tomura, M. *et al.* Monitoring cellular movement in vivo with photoconvertible fluorescence protein "Kaede" transgenic mice. *Proc. Natl. Acad. Sci.* **105**, 10871–10876 (2008).

17. Kitano, M. *et al.* Bcl6 protein expression shapes pre-germinal center B cell dynamics and follicular helper T cell heterogeneity. *Immunity* **34**, 961–972 (2011).

18. Allen, C. D. C., Okada, T. & Cyster, J. G. Germinal-Center Organization and Cellular Dynamics. *Immunity* **27**, 190–202 (2007).

19. Chan, T. D. & Brink, R. Affinity-based selection and the germinal center response. *Immunol. Rev.* **247**, 11–23 (2012).

20. Turner, J. S., Marthi, M., Benet, Z. L. & Grigorova, I. Transiently antigen-primed B cells return to naive-like state in absence of T-cell help. *Nat. Commun.* **8**, 15072 (2017).

21. Kislitsyn, A., Savinkov, R., Novkovic, M., Onder, L. & Bocharov, G. Computational Approach to 3D Modeling of the Lymph Node Geometry. *Computation* **3**, 222–234 (2015).

22. Grajdeanu, A. & Grajdeanu, A. MODELING DIFFUSION IN A DISCRETE ENVIRONMENT.

23. Lin, F. & Butcher, E. C. Modeling the Role of Homologous Receptor Desensitization in Cell Gradient Sensing. *J. Immunol. Baltim. Md 1950* **181**, 8335–8343 (2008).

24. Wu, D. & Lin, F. Modeling Cell Gradient Sensing and Migration in Competing Chemoattractant Fields. *PLoS ONE* **6**, e18805 (2011).

25. Gong, C. *et al.* Predicting lymph node output efficiency using systems biology. *J. Theor. Biol.* **335**, 169–184 (2013).

26. Bocharov, G. *et al.* Reaction-Diffusion Modelling of Interferon Distribution in Secondary Lymphoid Organs. *Math. Model. Nat. Phenom.* **6**, 13–26 (2011).

27. Guo, Z., Sloot, P. M. A. & Tay, J. C. A hybrid agent-based approach for modeling microbiological systems. *J. Theor. Biol.* **255**, 163–175 (2008).
28. Rot, A. & von Andrian, U. H. Chemokines in innate and adaptive host defense: basic chemokinese grammar for immune cells. *Annu. Rev. Immunol.* **22**, 891–928 (2004).
29. Bennett, L. D., Fox, J. M. & Signoret, N. Mechanisms regulating chemokine receptor activity. *Immunology* **134**, 246–256 (2011).
30. Press, W. H., Teukolsky, S. A., Vetterling, W. T. & Flannery, B. P. *Numerical Recipes 3rd Edition: The Art of Scientific Computing*. (Cambridge University Press, 2007).
31. Evans, S. *et al.* ASPASIA: A toolkit for evaluating the effects of biological interventions on SBML model behaviour. *PLOS Comput. Biol.* **13**, e1005351 (2017).
32. Maiuri, P. *et al.* Actin Flows Mediate a Universal Coupling between Cell Speed and Cell Persistence. *Cell* **161**, 374–386 (2015).
33. Luke, S., Cioffi-Revilla, C., Panait, L., Sullivan, K. & Balan, G. MASON: A Multiagent Simulation Environment. *SIMULATION* **81**, 517–527 (2005).
34. Sommerville, I. *Software Engineering*. (Pearson, 2010).
35. Alden, K. *et al.* Spartan: a comprehensive tool for understanding uncertainty in simulations of biological systems. *PLoS Comput. Biol.* **9**, e1002916 (2013).
36. Monroe, J. G. & Cambier, J. C. Sorting of B lymphoblasts based upon cell diameter provides cell populations enriched in different stages of cell cycle. *J. Immunol. Methods* **63**, 45–56 (1983).
37. Zigmond, S. H. Consequences of chemotactic peptide receptor modulation for leukocyte orientation. *J. Cell Biol.* **88**, 644–647 (1981).
38. Herzmark, P. *et al.* Bound attractant at the leading vs. the trailing edge determines chemotactic prowess. *Proc. Natl. Acad. Sci. U. S. A.* **104**, 13349–13354 (2007).
39. Sh, Z., Sj, S. & Da, L. Kinetic analysis of chemotactic peptide receptor modulation., Kinetic analysis of chemotactic peptide receptor modulation. *J. Cell Biol. J. Cell Biol.* **92, 92**, 34, 34–43 (1982).
40. Barroso, R. *et al.* EBI2 regulates CXCL13-mediated responses by heterodimerization with CXCR5. *FASEB J. Off. Publ. Fed. Am. Soc. Exp. Biol.* **26**, 4841–4854 (2012).
41. Tilo Beyer, M. M.-H. Modeling emergent tissue organization involving high-speed migrating cells in a flow equilibrium. *Phys. Rev. E Stat. Nonlin. Soft Matter Phys.* **76**, 021929 (2007).

42. Luther, S. A. *et al.* Differing Activities of Homeostatic Chemokines CCL19, CCL21, and CXCL12 in Lymphocyte and Dendritic Cell Recruitment and Lymphoid Neogenesis. *J. Immunol.* **169**, 424–433 (2002).

43. Gunn, M. D. *et al.* A B-cell-homing chemokine made in lymphoid follicles activates Burkitt's lymphoma receptor-1. *Nature* **391**, 799–803 (1998).

44. Wang, Y. & Irvine, D. J. Convolution of chemoattractant secretion rate, source density, and receptor desensitization direct diverse migration patterns in leukocytes. *Integr. Biol. Quant. Biosci. Nano Macro* **5**, 481–494 (2013).

45. Phair, R. D. & Misteli, T. Kinetic modelling approaches to in vivo imaging. *Nat. Rev. Mol. Cell Biol.* **2**, 898–907 (2001).

46. Read, M. N. Statistical and Modelling Techniques to Build Confidence in the Investigation of Immunology through Agent-Based Simulation. (University of York, 2011).

47. Vargha, A. & Delaney, H. D. A Critique and Improvement of the 'CL' Common Language Effect Size Statistics of McGraw and Wong. *J. Educ. Behav. Stat.* **25**, 101–132 (2000).

48. Cosgrove, J. *et al.* Agent-Based Modeling in Systems Pharmacology. *CPT Pharmacomet. Syst. Pharmacol.* n/a-n/a (2015) doi:10.1002/psp4.12018.
